# Morphology and autofluorescence of the thallus of the golden-brown alga *Hydrurus foetidus* by optical and fluorescence lifetime imaging

**DOI:** 10.1242/bio.062873

**Published:** 2026-10-30

**Authors:** Jens Ådne Rekkedal Haga, Live Lislerud Vestli, Kjetill Sigurd Jakobsen, Julien Alain Andre Resseguier, Felix Margadant, Oddmund Bakke, Xian Hu

**Affiliations:** Department of Biosciences, University of Oslo, 0316 Oslo, Norway

**Keywords:** Chrysophyceae, Confocal microscopy, FLIM, Autofluorescence, Tissue architecture, Rheophyte

## Abstract

The chrysophyte *Hydrurus foetidus* belongs to the psychrophilic, rheophilic algae of cold, fast-flowing glacial and alpine streams but remains poorly characterised at the cellular level. We present a systematic optical microscopy study of field-collected material of this organism, combining confocal imaging, quantitative morphometry, three-dimensional volumetry and fluorescence lifetime imaging (FLIM). Confocal imaging revealed a pseudoparenchymatous architecture: elongated, longitudinally aligned cells dominate the central axis, whereas round cells occupy the peripheral branches, and the two populations differ in volume. High-magnification imaging showed a consistent apical bias in nuclear position along the cell files of the central axis. FLIM of endogenous autofluorescence yielded reproducible lifetime signatures across the green, orange and far-red emission channels; in the far-red emission channel, the chlorophyll a signal resolved into a two-component mixture, and the round-cell population was bimodal in lifetime whereas the elongated population was not. All material derives from a single winter collection; the whole-thallus spatial analyses come from one intact thallus, while morphometry, volumetry and FLIM sampled cells from multiple thalli of that collection, and the data are analysed accordingly. Together, these data provide a morphological and photophysical foundation, and a label-free autofluorescence reference, for future cell-biological studies of *H. foetidus* and of multicellular organisation among the chrysophytes.

## Teaser

**Summary:** Label-free confocal and fluorescence lifetime imaging reveals position-dependent cell shape, size and nuclear polarity in the stream alga *Hydrurus foetidus* and provides an autofluorescence lifetime reference.

## INTRODUCTION

*Hydrurus foetidus* (Villars) Trevisan is a golden-brown chrysophyte alga that forms conspicuous, gelatinous, arbuscular thalli in cold, fast-flowing glacial and alpine streams, where it can dominate the benthic community and shape invertebrate distribution (Moog and Janecek, 1991; Klaveness, 2017, 2019). This species has attracted attention since the 19th century because its macroscopic, differentiated thallus is exceptional among chrysophytes, and early researchers documented its development, branching and life cycle in detail (Lagerheim, 1888; Molisch, 1918; Szklarczyk, 1953; Hovasse and Joyon, 1960). Molecular analyses of the 18S and 28S rDNA have since confirmed its placement within the Chrysophyceae and clarified the identity and phylogeny of the genus (Klaveness et al., 2011; Hao et al., 2024), and the species has been established in laboratory culture by Klaveness and Lindstrøm (2011); all material examined in the present study, however, was field collected and fixed on site, and no cultured material was used. Classical electron-microscopy studies have established much of what is known of its cell biology, including the distinctive tetrahedral zoospore with its elaborate microtubular skeleton, the organisation of mitosis and cell division, and the occurrence of virus-like particles (Wujek, 1969; Hoffman, 1978; Vesk et al., 1984; Hoffman et al., 1986). A draft genome and transcriptome data are available (Bråte et al., 2019), as well as two new long-read genome assemblies (Francomme, 2025; GenBank accession number GCA_978795795.1), yet basic cell-level features such as tissue architecture, nuclear organisation and autofluorescence remain largely uncharacterised by modern optical microscopy.

Advanced fluorescence microscopy, including FLIM, provides powerful tools for a morphological description that also captures autofluorescence properties in both the spectral and the time domains. FLIM resolves the photophysical properties of fluorescent molecules independently of their local concentration or excitation intensity, making it particularly suited to algal specimens in which intense, spectrally broad autofluorescence from photosynthetic pigments (chlorophylls a, c1 and c2, and carotenoids including fucoxanthin and other xanthophylls) and metabolic cofactors [NAD(P)H, flavins] routinely overwhelms intensity-based measurements. Characterising the molecules that underlie this complex autofluorescence is a prerequisite for future cell-biological study of the organism using fluorescence microscopy. Here, we combine confocal morphometry, three-dimensional volumetry and multichannel FLIM to provide that baseline for *H. foetidus*. We deliberately sampled the organism in its natural habitat (Fig. 1) because the psychrophilic, rheophilic growth form and the differentiated thallus architecture that motivate this work develop in fast-flowing cold streams and are not reproduced in culture.

## RESULTS AND DISCUSSION

### Pseudoparenchymatous tissue architecture and mechanobiological adaptations

Confocal imaging of the dissected thallus fragments revealed a reproducible, spatially segregated pattern of cell morphology. The cells within the peripheral branch tissue – referred to here as round cells, corresponding to the ‘branch cells’ of Klaveness and Lindstrøm (2011) – were consistently spherical to ovoid, approximately 8 μm across (median in-plane width 8.3 μm; equivalent-sphere diameter 8.0 μm derived from the three-dimensional volumes below) and arranged in a disordered configuration lacking a preferred direction of elongation. These dimensions are consistent with those reported in the literature on wild material, in which cell sizes of 8–16×12–22 μm (Mack, 1953), 7–20 μm (Joyon, 1963) and 5–15 μm (Vesk et al., 1984) have been reported and, allowing for the larger cell sizes of cultured material, with the ‘branch cells’ (intercalary and free branch cells combined, 12–13 μm on average) of cultured *H. foetidus*; cultured ‘central axis cells’, by contrast, are ellipsoid and roughly twice as long as they are wide (mean 25×10 μm; Klaveness and Lindstrøm, 2011), the same shape contrast we observe between the two morphotypes. In marked contrast, the core of the main axis of the thallus was dominated by highly elongated cells (the ‘central axis cells’ of Klaveness and Lindstrøm, 2011). These elongated cells displayed a pronounced longitudinal alignment, running parallel to one another in tightly packed arrays along the main axis of the thallus (Fig. 2A–C). Examination of fresh material indicates that elongated cells do not occur in the branches, nor round cells in the central axis (J.Å.R.H., unpublished), so cell shape is a reliable indicator of position; we nevertheless use the shape-based terms because, in the fragment-derived datasets below, cells were classified by morphology and their position within the thallus was not recorded directly. At single-cell resolution, the two morphotypes are equally distinct: elongated cells show an ovoid profile in which the nucleus and the plastid occupy separate positions along the long axis (Fig. 2F–H), whereas round cells show a compact profile with a cortical plastid (Fig. 2I–K). The contrast is quantitative as well as qualitative: in 20× brightfield fields, cells within dense strands were more elongated than cells in the surrounding loose matrix (median aspect ratio 1.51, *n*=65, versus 1.22, *n*=24; Mann–Whitney *P*=6.9×10^−5^; Fig. 2D), and across 2422 segmented confocal cells, the median aspect ratio was 1.97.

**Fig. 1.**
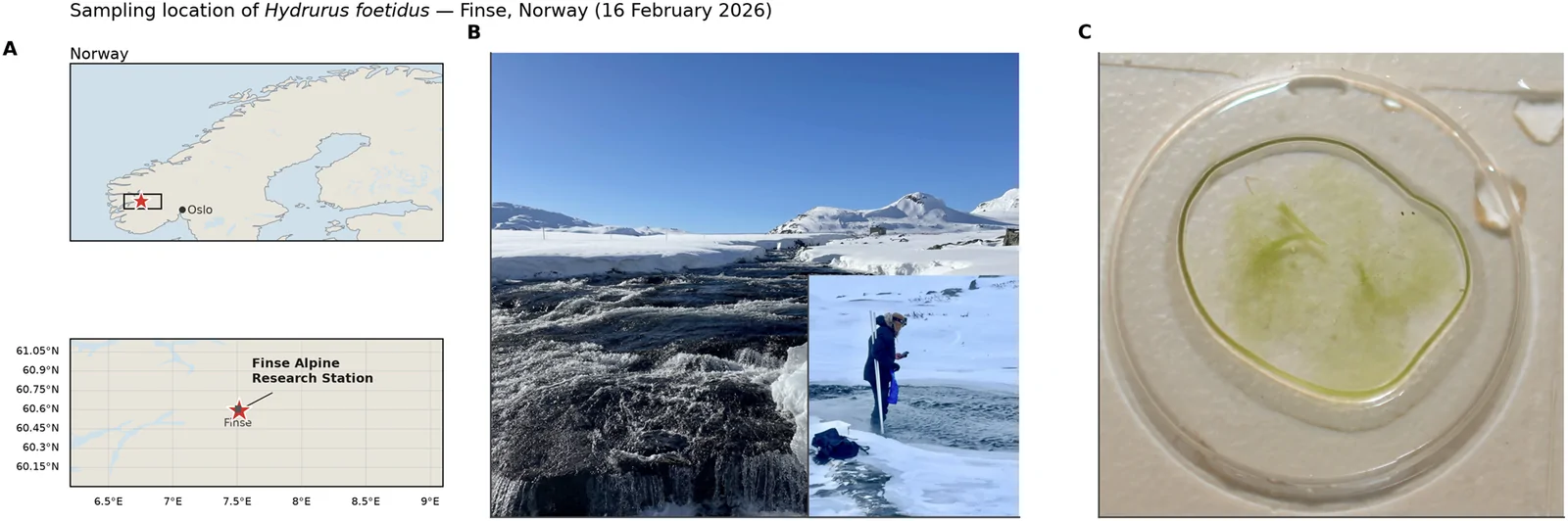
**Sampling location of *Hydrurus foetidus*.** (A) Locator map showing the collection site next to the Finse Alpine Research Station (University of Oslo; star, 60.594° N, 7.515° E) on the north-western Hardangervidda plateau, south-central Norway (∼1220 m a.s.l.), with a regional inset below the national map. (B) The stream at the collection site next to the Finse Alpine Research Station, photographed on an earlier visit (14 March 2025); inset, sampling from the ice-free channel of the stream on the day of collection (16 February 2026). (C) A field-collected, fixed *H. foetidus* thallus mounted in a 35 mm glass-bottom imaging dish (MatTek) for microscopy.

**Fig. 2.**
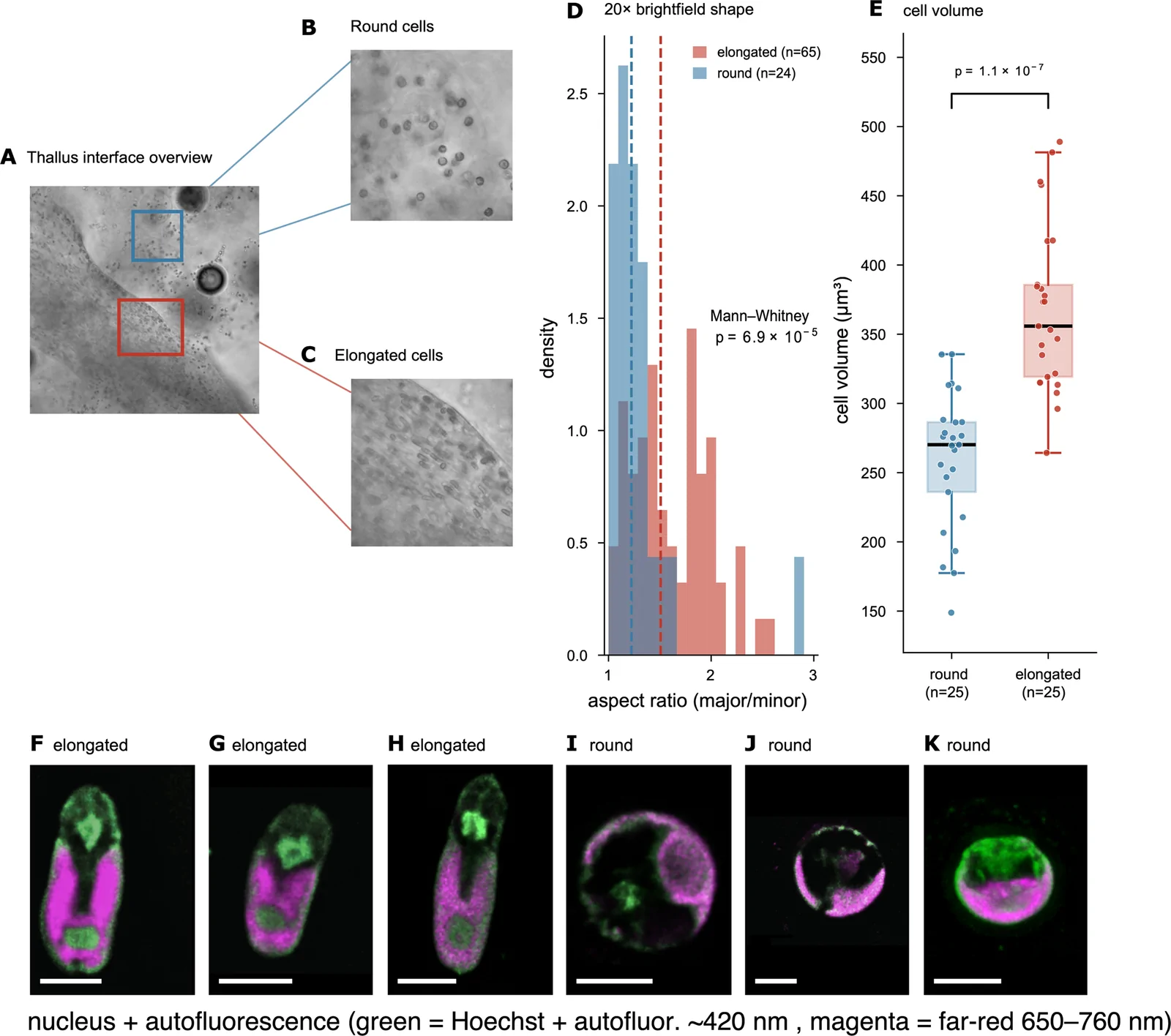
**Cell morphology of *Hydrurus foetidus* varies with position in the thallus.** (A) Brightfield overview of a thallus interface between a dense central strand and the surrounding looser matrix (20×/0.8 NA air objective); coloured boxes mark the regions enlarged in B (blue, loose matrix) and C (red, dense strand). (B) Enlarged loose-matrix region, where cells are predominantly round, and (C) enlarged dense-strand region, where cells are predominantly elongated. (D) Distribution of cell aspect ratio (major/minor axis) from 20× brightfield images: cells in dense-strand regions (labelled ‘elongated’; *n*=65, median 1.51) are more elongated than cells in the loose matrix (labelled ‘round’; *n*=24, median 1.22); dashed lines mark group medians (Mann–Whitney *P*=6.9×10^−5^). (E) Three-dimensional cell volumes from maximum-likelihood-estimation-deconvolved confocal image stacks: elongated cells occupy a larger volume (*n*=25, median 356 μm^3^) than round cells (*n*=25, median 270 μm^3^; Mann–Whitney *P*=1.1×10^−7^); boxes show the interquartile range, the black line shows the median, and the whiskers show the most extreme values within 1.5× the interquartile range, with all individual cells plotted. (F–K) Representative 60×/1.30 NA silicone-immersion confocal images of individual cells: elongated cells from strand tissue (F–H) and round cells from looser tissue (I–K). Green, Hoechst and autofluorescence in the ∼420 nm range; magenta, plastid autofluorescence in the far-red (650–760 nm) range. Scale bars: 5 μm.

This spatial segregation resembles the pseudoparenchymatous medulla–cortex organisation described in macroscopic marine brown algae (Phaeophyceae), and in *H. foetidus*, it has been noted in classical accounts of the thallus structure (Hovasse and Joyon, 1960; Klaveness, 2019). Berthold (1878, as *Hydrurus penicillatus*) illustrated two elongated cells from the interior of the thallus; one of these cells may represent a central axis cell, although his figures and descriptions are limited; Mack (1953) described greatly enlarged cells, on average about 20 μm wide and 32 μm long, that occur exclusively inside older parts of the thallus and are not interspersed among the small peripheral cells. Earlier reports of elongated cells outside the central axis, or of unpolarised central cells, may reflect differences in fixation and preservation. Because the Phaeophyceae are a separate class from the chrysophytes, this resemblance reflects convergent evolution rather than shared ancestry. *H. foetidus* lacks a rigid cellulosic cell wall, yet it thrives in rheophilic, highly turbulent glacial streams. By compressing and aligning elongated cells down the central axis, the alga forms a dense, fibrous core that may function as a structural ‘cable’ or ‘tendon’. Such a centralised bundle could favour tensile strength, allowing the thallus to withstand continuous hydrodynamic drag without tearing away from its anchoring surface. Conversely, the round cells of the peripheral branches fan outward into a loosely ordered arrangement. This architectural transition expands the surface area of the thallus, minimising self-shading while maximising exposure to light and to fast-flowing, CO_2_- and oxygen-rich water. The resulting irregular canopy may also disrupt uniform flow, converting linear forces into microscopic eddies that mitigate mechanical stress at the attachment base. Comparable structural trade-offs are well characterised in wave-swept macroalgae, where thallus flexibility, reconfiguration under load and branching architecture together set the hydrodynamic drag a thallus experiences and where added branches can increase drag while simultaneously increasing the tissue cross section available to bear it (Martone et al., 2012; Starko et al., 2015). Those studies concern intertidal Phaeophyceae under oscillatory wave loading rather than steady, unidirectional lotic flow, so the parallel is one of design principle rather than of matched hydrodynamic regime; it nonetheless establishes that an axial-core-plus-peripheral-canopy arrangement of the kind described here is the sort of architecture that measurably alters drag and that the hypotheses above are testable using the established macroalgal biomechanical toolkit – tensile testing of excised thallus segments and drag measurement of intact thalli in a flow tank. We emphasise that these mechanical interpretations are hypotheses derived from morphology alone and remain to be tested by direct biomechanical measurement.

Three-dimensional volumetry showed that this shape dimorphism is accompanied by a difference in cell size: elongated cells occupied a significantly larger volume than peripheral round cells (medians 356 versus 270 μm^3^; *n*=25 per class; Mann–Whitney *P*=1.1×10^−7^, rank-biserial *r*=0.88; Fig. 2E). The enlargement was driven by axial extension rather than isotropic growth: elongated cells were approximately 1.8 times longer than round cells (medians 15.6 versus 8.7 μm) but had a smaller (∼0.7×) cross-sectional area (median width 6.9 versus 8.3 μm), so that lengthening more than offset their narrower girth. In dimensionless terms, in-plane elongation differed strongly between morphotypes (medians 2.43 versus 1.07; Mann–Whitney *P*=1.4×10^−9^), whereas sphericity was similar (0.61 versus 0.63; *P*=0.59), indicating that the volume difference arises from axial extension rather than surface elaboration; being scale free, both measures are insensitive to any uniform fixation-induced volume change. Several non-mutually exclusive processes could be responsible for coupling elongation to this increase in volume. First, the two populations may occupy different points along a growth-and-maturation trajectory: if the peripheral, round cells represent a younger, less differentiated state, and the elongated cells of the central axis represent a more mature state, then it would be expected that cell volume would increase as the cells commit to axial growth within the core. Second, the enlargement is consistent with vacuole- and storage-driven anisotropic expansion, as the elongated cells are largely occupied by the massive vacuoles and chrysolaminarin energy-storage bodies described below. Turgor-driven expansion of such compartments along a single axis is an economical way to increase volume as it adds relatively little membrane surface area. This is consistent with the modestly lower surface-to-volume ratio of the elongated population (medians 1.12 versus 1.21 μm^−1^; Mann–Whitney *P*=0.046, a difference close to the conventional significance threshold and therefore only suggestive). Third, the size difference may be a structural corollary of the load-bearing role proposed above: longer cells form longer continuous fibres with fewer transverse cell junctions, and hence fewer mechanical discontinuities, per unit length of the thallus ‘cable’, so that mechanical optimisation and cell enlargement would be expected to occur together in the core. Distinguishing between these possibilities will require mapping cell volume against radial and developmental position, as well as quantifying vacuolar and storage-body content directly.

### Unidirectional cellular polarity and organelle positioning

High-magnification confocal analysis of Hoechst-stained elongated cells revealed a consistent organelle positioning system. Rather than exhibiting the stochastic nuclear positioning observed in many unpolarised cells, the nuclei of *H. foetidus* were displaced towards one end of the elongated-cell profile. Quantification across the whole thallus (Fig. 3A–C) confirmed this bias: nuclei were displaced a median of 46% of the way towards a cell pole (median displacement 2.5 μm), and 85% of cells were displaced towards the same pole when the displacement was projected onto the thallus long axis (median signed projection −2.2 μm; *n*=2422 cells, descriptive; Fig. 3B). Because cells within one thallus are not independent, statistical inference was performed with the imaging region of interest (ROI) as the unit of replication (ROI positions shown in Fig. 3C): all 15 ROIs showed the same direction of bias (binomial *P*=6.1×10^−5^; Wilcoxon test on ROI means *P*=6.1×10^−5^; mean of ROI means −1.88 μm, s.d. 0.57; Fig. 3B). The same cells were strongly co-aligned with the thallus axis (nematic order parameter S=0.79; median angular deviation 11.9°; Rayleigh test on doubled angles, *P*<0.001), and this alignment was essentially uniform across the three sampled zones (S=0.80, 0.79 and 0.78 for margin, mid and core, respectively; Fig. 3D). The nuclear bias was therefore coordinated across the cell files of the central axis, with the nuclei consistently offset towards the growing apical direction of the thallus. The same organisation is visible at the distal branch endings, where clustered rounded–ovoid cells retain pole-displaced nuclei (Fig. 3E). The volumetry stacks provide an independent, three-dimensional confirmation of this nuclear positioning: in the subset acquired with a per-cell Hoechst z-stack (13 elongated and two round cells, from the acquisition batch excluded from the volume comparison because of its cortical far-red labelling, which does not affect the nuclear channel), the elongated-cell nucleus (equivalent diameter≈2.5 μm) lay a median of 1.8 μm from the cell centre along the major axis (median fractional offset 0.41 of the half-length), in good agreement with the mosaic-derived cell-level displacement (median 2.5 μm; 46% of half-cell length). Too few round-cell nuclear stacks were available for a morphotype comparison, and the two round cells are reported descriptively in Table S1, which lists the per-cell measurements.

**Fig. 3.**
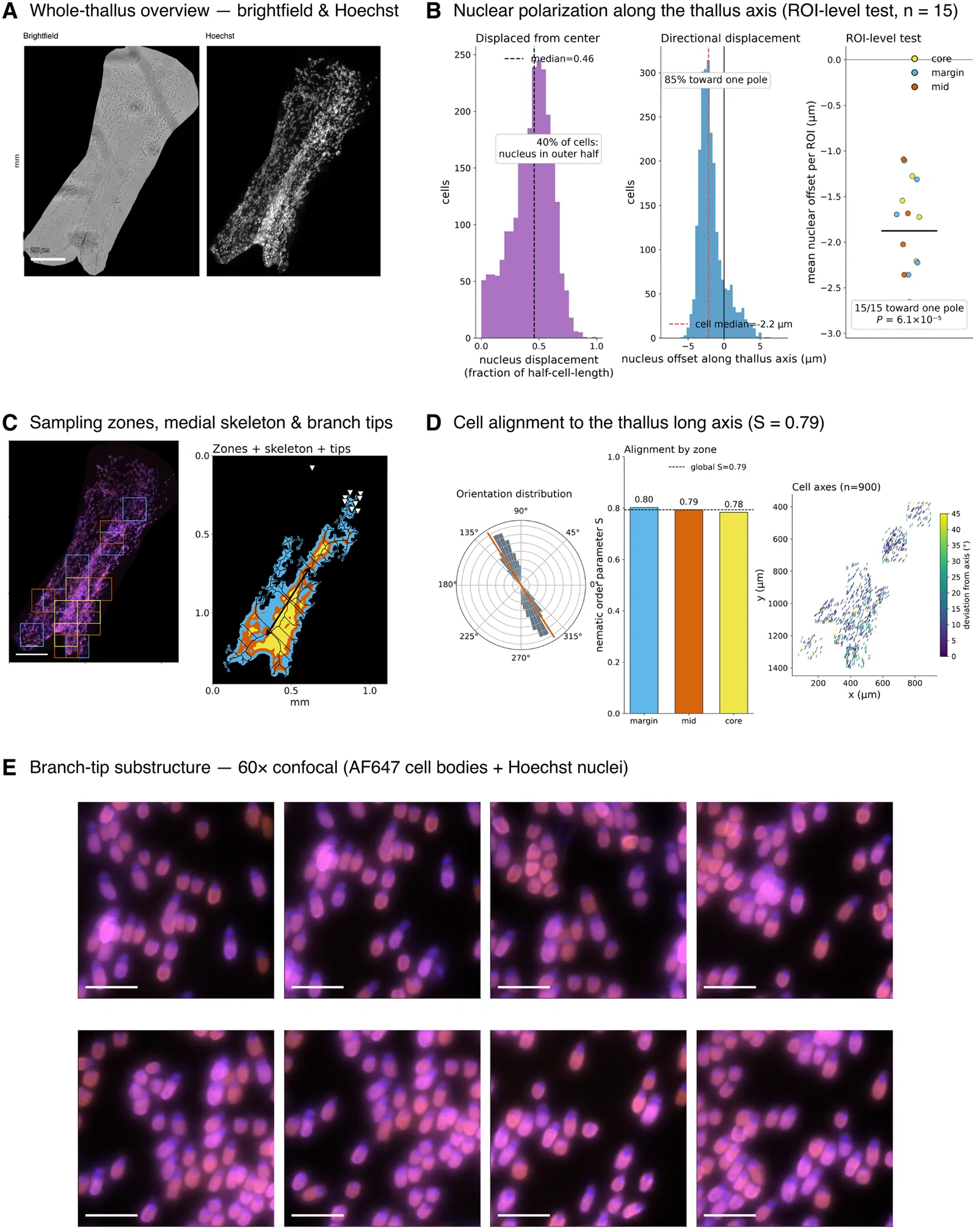
**Spatial organisation of the *Hydrurus foetidus* thallus (2422 segmented confocal cells).** (A) Whole-thallus overview showing brightfield structure (left) and Hoechst-stained nuclei (right). Scale bar: 200 μm. (B) Nuclear polarisation along the thallus axis. Left (‘Displaced from center’): nucleus displacement as a fraction of half-cell length; the dashed line marks the median (0.46), and 40% of cells have the nucleus in the outer half of the cell. Middle (‘Directional displacement’): signed nucleus offset along the thallus long axis; the red dashed line marks the cell-level median (−2.2 μm), with 85% of cells displaced towards the same pole (descriptive values; single thallus). Right (‘ROI-level test’): inference with the imaging ROI as the unit of replication; each point is the mean nuclear offset of one ROI (blue, margin; orange-red, mid; yellow, core), the black bar marks the mean of ROI means (−1.88 μm, s.d. 0.57), and all 15 ROIs are biased towards the same pole (binomial *P*=6.1×10^−5^; Wilcoxon test on ROI means *P*=6.1×10^−5^). (C) Left: confocal overlay with the 15 sampling ROIs colour coded by zone (blue, margin; orange-red, mid; yellow, core). Scale bar: 200 μm. Right: zone map (same colour code) with the medial skeleton (black), detected branch tips (white arrowheads) and the fitted thallus long axis (black double-headed arrow); axes in mm. (D) Cell alignment to the thallus long axis. Left: cell orientation distribution, with the thallus long axis shown in orange-red. Middle: nematic order parameter S per zone (S=1, perfectly aligned; S=0, random); the dashed line marks the global S=0.79 (margin 0.80, mid 0.79, core 0.78; Rayleigh test on doubled angles, *P*<0.001). Right: individual cell axes across the thallus (*n*=900 cells shown), colour coded by angular deviation from the thallus long axis (0–45°). (E) Branch-tip substructure: eight representative 60× confocal fields of distal branch tips (AF647 cell-body autofluorescence, red; Hoechst nuclei, blue) showing clustered rounded–ovoid cells with pole-displaced nuclei. Scale bars: 20 μm.

This degree of unidirectional polarity is consistent with an underlying, coordinated apical–basal tissue organisation. The reproducible positioning of the nucleus suggests the presence of an internal anchoring system. In flagellated chrysophyte lineages, the nucleus is typically tethered to the basal bodies by a microtubular network (Wujek, 1969; Vesk et al., 1984; Hoffman et al., 1986). This cytoplasmic scaffold may prevent organelles from drifting, helping to maintain structural integrity when the thallus is subjected to stream-induced mechanical swaying. This physical anchoring may be further reinforced by space-constricting intracellular components. The proximal and central cytoplasm is largely occupied by massive vacuoles and chrysolaminarin energy-storage bodies, which crowd the nucleus towards the apical pole. We hypothesise that maintaining the nucleus at a uniform, shielded position protects it from shear-induced displacement while aligning the cell's longitudinal axis with the primary direction of growth. Because these observations derive from fixed, single-time-point material, a strict developmental interpretation of apical–basal polarity would require time-resolved imaging or fate markers. The present data establish a robust, quantified positional bias rather than demonstrating a developmental axis.

### Autofluorescence lifetime profiling by FLIM

Because *H. foetidus* features an intense, wide-spectrum autofluorescence background – driven by its light-harvesting pigments (chlorophylls a, c1 and c2, together with the accessory carotenoid fucoxanthin characteristic of the Chrysophyceae; Ricketts, 1965) and by metabolic cofactors such as NAD(P)H and flavins – conventional intensity-based imaging reaches its limits. We therefore applied time-resolved FLIM to add an independent, label-free contrast dimension, characterising fluorescence decay across the green (λem 498–551 nm), orange (571–630 nm) and far-red (650–760 nm) emission channels. Because chlorophyll a is the dominant far-red emitter, whereas chlorophyll c and the carotenoids absorb and emit at shorter wavelengths, the far-red channel reports primarily on the chlorophyll a pool, while the green and orange channels capture the broader accessory-pigment and cofactor background. Endogenous chlorophyll fluorescence lifetime has been exploited in this way in several other algal systems, and these provide the closest available methodological benchmarks for the present work. In *Chlamydomonas reinhardtii*, FLIM of the slow chlorophyll fluorescence transient distinguished non-photochemical-quenching mutants from wild type and responded predictably to photosynthetic inhibitors (Holub et al., 2007); in *Tetraselmis*, chlorophyll a lifetime tracked reversible UV-induced changes in photosynthetic activity (Kristoffersen et al., 2016); and in *Chlorella*, time-resolved chlorophyll fluorescence proved sensitive to intracellular pH (Marcek Chorvatova et al., 2020). All three are green algae belonging to the chlorophyte group and have a chlorophyll a/b light-harvesting antenna, whereas *H. foetidus* is a chrysophyte containing chlorophyll a/c and fucoxanthin. Therefore, absolute lifetimes cannot be transferred directly between these species and our data. What is transferable is the underlying chlorophyll a photophysics, together with the central methodological point these studies establish: that lifetime, unlike intensity, reports on the functional state of the pigment pool rather than on its abundance and is therefore interpretable in a specimen whose absolute fluorescence intensity is uncontrolled. Phasor analysis confirmed that these endogenous signatures occupy well-defined, reproducible domains, with the far-red channel yielding the most robust and consistent signal (Fig. 4). Across all cells, the median lifetimes were 1.67 ns (far red), 1.15 ns (orange) and 1.19 ns (green).

**Fig. 4.**
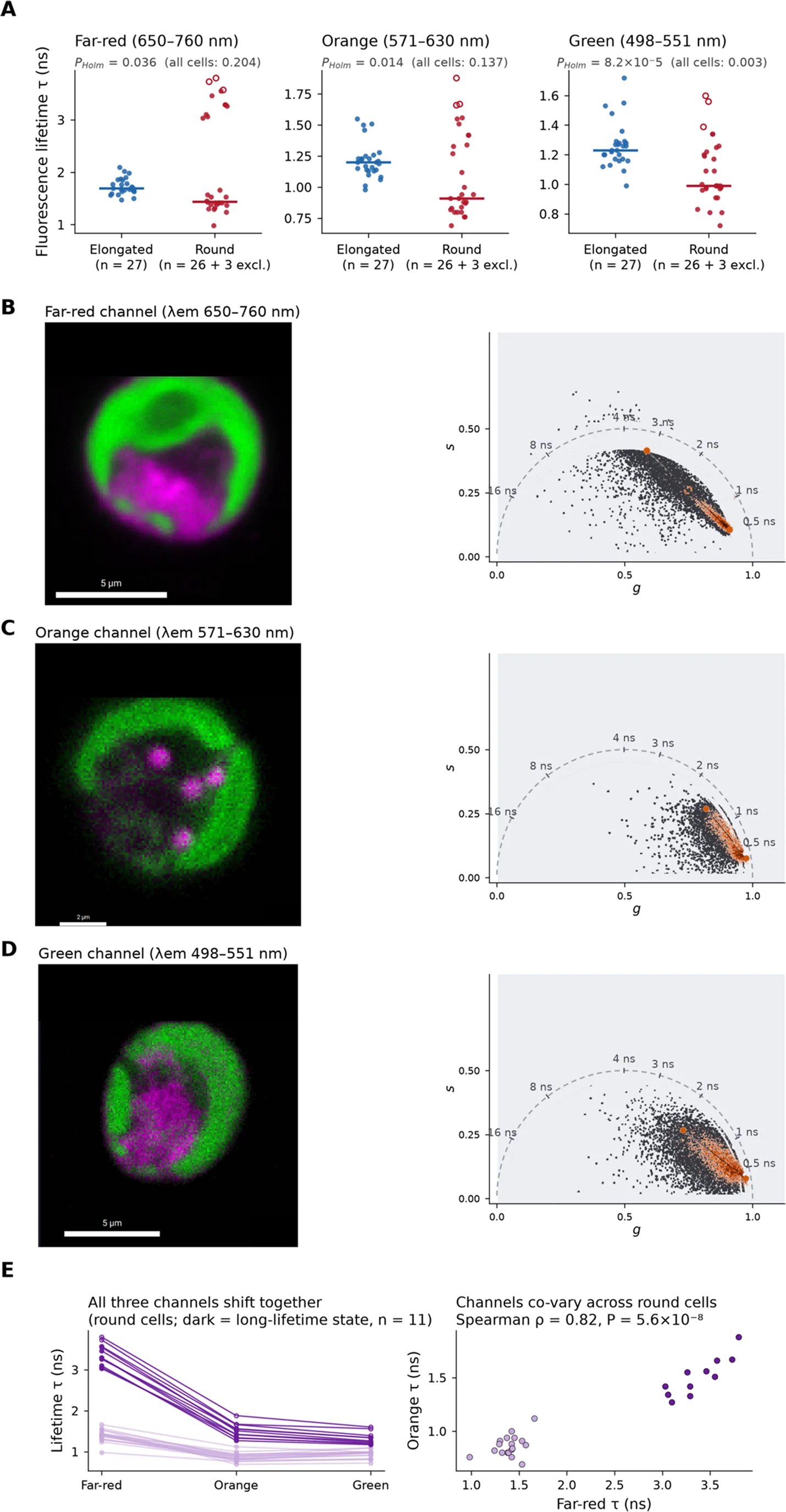
**Fluorescence lifetime imaging (FLIM) of *H. foetidus* autofluorescence.** (A) Mean fluorescence lifetime (τ, ns) of elongated (*n*=27) versus round cells in each of the three emission channels: far red (λem 650–760 nm), orange (λem 571–630 nm) and green (λem 498–551 nm). Each point is one cell; filled circles, primary dataset (*n*=26 round); open circles, the three acquisition-flagged round cells excluded from the primary analysis and shown for transparency; horizontal bars mark primary-dataset medians. Holm-corrected Mann–Whitney *P*-values are given for the primary analysis, with the all-cell sensitivity analysis in parentheses (far red 0.036 versus 0.204; orange 0.014 versus 0.137; green 8.2×10^−5^ versus 0.003). Round-cell far-red lifetimes are bimodal (Hartigan's dip test *P*=6.9×10^−4^; components at 1.40±0.15 ns, *n*=18 and 3.38±0.27 ns, *n*=11; robust to the exclusion, *P*=6.4×10^−3^), whereas elongated-cell lifetimes are not (*P*=0.98). (B–D) Representative phase-segmented FLIM overlays (left) and corresponding phasor plots (right) for a single cell in each channel: (B) far-red channel, (C) orange channel and (D) green channel. In the overlays, green and magenta encode the two lifetime components resolved by phasor segmentation (short- and long-lifetime populations); the phasor plots show the pixel distribution (dark points) with the two selected component positions (orange markers) and the universal semicircle with reference lifetimes (0.5–16 ns). For legibility, the phasor plots were re-rendered from the acquisition-software display: the colour scheme was inverted from the native dark background to a light background, and the reference semicircle, lifetime labels and g/s axes were redrawn as vector graphics; the pixel distribution, component positions and trajectory are the original, unaltered data. Scale bars: 2–5 μm as indicated. (E) The long-lifetime state is a coherent, cell-level state: left, per-cell lifetimes across the three channels for round cells (dark, long-lifetime state, *n*=11; light, short-lifetime state, *n*=18; open symbols, acquisition-flagged cells); right, far-red versus orange lifetimes co-vary across round cells (Spearman ρ=0.82, *P*=5.6×10^−8^).

To probe cellular heterogeneity, we compared per-cell mean lifetimes between elongated (‘long’, *n*=27) and round cells across all three channels (Fig. 4A). Three round cells whose acquisitions were flagged during recording, before any analysis, were excluded from the primary comparison based on this pre-specified acquisition-quality criterion (primary *n*=26 round; the all-cell analysis, *n*=29, is reported as a sensitivity check), and *P*-values were Holm corrected across the three channels. In the primary analysis, all three channels differed significantly between morphotypes, with longer lifetimes in elongated cells (Holm-corrected *P*=0.036 far red, 0.014 orange, 8.2×10^−5^ green; medians 1.69 versus 1.44, 1.20 versus 0.91 and 1.23 versus 0.99 ns, respectively). In the all-cell sensitivity analysis, the green channel remained significant (Holm-corrected *P*=0.003) whereas the far-red and orange channels did not (*P*=0.20 and 0.14), so the channel-median comparisons – though not the bimodality below – depend on the exclusion, and we report both throughout. Beyond the medians, the far-red distributions differed in shape: elongated cells formed a single, tight lifetime cluster, whereas round cells separated into two groups – one overlapping with the elongated population at 1.40±0.15 ns (mean±s.d., *n*=18) and a second, markedly longer-lived group at 3.38±0.27 ns (*n*=11, of which eight were unflagged), with no round cell falling between 1.66 and 3.03 ns. Hartigan's dip test rejected unimodality for the round-cell far-red lifetimes (D=0.121, *P*=6.9×10^−4^) but not for the elongated cells (D=0.043, *P*=0.98), and a two-component Gaussian mixture was strongly preferred over a single component for the round cells (ΔBIC=50.6) but not for the elongated cells (ΔBIC=−5.8, favouring one component). The bimodality is robust to the acquisition-quality exclusion: with the three flagged cells removed, the long-lifetime mode retains eight cells (3.03–3.55 ns), the dip test still rejects unimodality (*P*=6.4×10^−3^) and the two-component mixture remains strongly preferred (ΔBIC=49.0). This bimodality within the round-cell population, rather than any shift in the group mean, is the most striking feature of the comparison.

The single-cell phasor plots indicate the origin of this heterogeneity. In the far-red channel, pixels fell along a straight line between two lifetime poles – a short-lived component (∼0.5 ns) and a long-lived component (∼3–4 ns) – the signature of a two-component mixture and, spatially, of two distinct compartments (Fig. 4B). The simplest interpretation attributes both poles to chlorophyll a in different photophysical states: the short-lived pole to photochemically quenched, functionally coupled chlorophyll a in active thylakoids, where open reaction centres rapidly drain excitation energy, and the long-lived pole to uncoupled, closed or degrading chlorophyll a, possibly together with storage-associated autofluorescence. This assignment is based on established chlorophyll a photophysics rather than on the present data alone. In Photosystem II preparations, chlorophyll a fluorescence lifetime distributions shift from being short lived when reaction centres are open and photochemically active to being substantially longer lived when they are closed (Govindjee et al., 1990), and *in vivo*, photoinactivated reaction centres are characterised by a distinct long-lifetime population (Matsubara and Chow, 2004). Lifetimes in the 3–4-ns range, as at our long-lived pole, approach the intrinsic unquenched lifetime of chlorophyll a and therefore indicate that the pigment is effectively decoupled from photochemistry, whereas the sub-nanosecond pole is in the range expected for functionally connected, photochemically quenched antenna. Two differences from those studies bound the comparison: our values are derived from chemically fixed material and represent phasor centre-of-mass lifetimes integrated over whole cells rather than decay-associated spectra of isolated complexes. The agreement should therefore be considered qualitative – in the direction and approximate magnitude of the short-to-long shift – rather than numerical. NAD(P)H and flavin emission, by contrast, falls in the blue and green–yellow rather than the far-red and therefore informs the green and orange channels rather than this far-red assignment. Consistent with a cell-level physiological state rather than a single-channel artefact, the 11 long-lifetime round cells were elevated in all three emission channels simultaneously (Holm-corrected *P*=3.7×10^−5^ for each channel), the channels co-varied strongly across round cells (far-red versus orange Spearman ρ=0.82, *P*=5.6×10^−8^; orange versus green ρ=0.97, *P*=8.7×10^−18^), the fold change was graded by wavelength (far red 2.3×, orange 1.8×, green 1.3×), and the high-lifetime state was confined to round cells (zero of 27 elongated cells versus 11 of 29 round cells above 3.0 ns; Fig. 4E). A detector, photon-statistics or focus artefact would not produce a wavelength-graded, cross-channel-coherent shift restricted to one morphotype.

Two properties of the preparation constrain all of these lifetime interpretations. First, the material was aldehyde fixed on site, and aldehyde fixation itself is a recognised source of fluorescence artefact: formaldehyde generates fluorescent condensation products that increase tissue autofluorescence background in confocal imaging (Baschong et al., 2001), and fixation can alter measured fluorescence lifetimes even for well-characterised fluorophores (Joosen et al., 2014). The lifetimes reported here are therefore properties of fixed *H. foetidus* under a defined protocol rather than of the living alga, and the absolute values should be treated as a reference for comparably fixed material, not as *in vivo* photophysical constants. Of the three channels, the far-red channel is the least exposed to this artefact because the chlorophyll a emission there is spectrally well separated from the blue-green region in which aldehyde-derived products emit – which is a further reason, alongside its signal quality, for resting the main photophysical argument on that channel. Second, and conversely, endogenous autofluorescence is a genuinely useful contrast mechanism in fixed plant and algal tissue, where it has been used to resolve anatomy across taxa from algae to angiosperms without any exogenous labelling (Pegg et al., 2021); the lifetime signatures reported here extend that label-free strategy from the spectral domain into the time domain. The extent to which the round-cell bimodality persists in living material remains to be seen.

Consistent with their bimodal lifetimes, round cells were also morphologically heterogeneous: a subset contained lipid-droplet-like inclusions with distinct far-red and orange lifetimes (Fig. 4B–D), whereas elongated cells appeared uniform. One interpretation is that round cells occupy a range of physiological or cell-cycle states, which would imply that active cell-cycle progression is largely confined to the round population while the elongated cells represent a more uniform, differentiated and potentially arrested state. This proposal remains speculative and would require direct testing, for example, by combining cell-cycle staging with FLIM. Independently of its outcome, these data establish label-free FLIM as a practical means of separating subcellular compartments in this pigment-rich alga – including the putative contractile vacuole, the chrysolaminarin storage vacuole and lipid droplets – providing a foundation for compartment-specific physiological studies.

### Conclusions and outlook

Together, these data provide a systematic quantitative optical microscopy and photophysical characterisation of the thallus of *H. foetidus*, based on material from a single February collection – with the whole-thallus spatial analyses from one intact specimen and cell-level datasets from multiple thalli whose identities were not tracked per cell; the seasonal and between-individual generality of these observations remains to be established. The data reveal a pseudoparenchymatous, tissue-like architecture defined by a clear spatial segregation of cell types – elongated, longitudinally aligned cells forming a structural core and unoriented round cells populating the peripheral branches – together with a reproducible apical bias in nuclear position along the cell files of the central axis. While the dense, gelatinous extracellular matrix and overlapping autofluorescence background present clear barriers to conventional intensity-based nuclear staining and imaging, we show that time-resolved FLIM resolves these signatures into distinct, interpretable phasor domains. Each of the hypotheses raised here is testable with a defined experiment compatible with the organism's field constraints (material must be treated on site and fixed before transport). Whether round and elongated cells represent different differentiation states could be resolved by on-site 5-ethynyl-2′-deoxyuridine (EdU) pulse-labelling of thallus fragments in stream water, followed by fixation and click detection with Hoechst counterstaining and scoring of S-phase cells by morphotype. To distinguish the two far-red lifetime poles as photophysical states of chlorophyll a, paired fragments from the same thallus could be incubated on site with and without the photosystem II inhibitor 3-(3,4-dichlorophenyl)-1,1-dimethylurea (DCMU), fixed at matched time points and imaged under identical FLIM settings; closing reaction centres should lengthen the short-lived component and compress the bimodal gap. Cytoskeletal anchoring of the nucleus can be investigated using anti-tubulin immunofluorescence on fixed material, with the caveat that tubulin epitope conservation in chrysophytes is untested. Finally, generalisation beyond the present specimen requires replication across thalli: a power analysis on the between-ROI variability of these data indicates that five to six thalli, sampled across at least two collection dates and stream positions and analysed with the thallus as the unit of replication, would suffice for the volume and polarisation comparisons. By establishing this baseline framework and identifying targeted protocol improvements – including far-red lifetime-based segmentation and enzymatic matrix permeabilisation – this work lays a morphological and biophysical foundation for further study of *H. foetidus* as a tractable system for cell organisation, polarity and mechanobiology among the stramenopiles – a lineage in which the transition to a differentiated multicellular thallus, analysed in depth for animals, plants and volvocine algae (Buss, 1987; Maynard Smith and Szathmáry, 1995; Michod, 1998), has so far received little cell-biological attention.

## MATERIALS AND METHODS

### Sample collection and preparation

Samples were collected next to the Finse Alpine Research Station (University of Oslo; 60.59364° N, 7.51479° E), located on the Hardangervidda plateau in south-central Norway, on 16 February 2026 (Fig. 1A,B). The samples were fixed on site in 4% paraformaldehyde (prepared from 16% methanol-free formaldehyde, Pierce/Thermo Scientific, cat. no. 28906) in a PHEM buffer solution (Electron Microscopy Sciences, via Fisher Scientific, cat. no. 50-193-1295). All material derives from this single collection. The whole-thallus confocal mosaic and the spatial analyses derived from it (Fig. 3) come from one intact thallus. This thallus was selected because it was the only specimen whose entire structure could be captured intact within practical acquisition limits; the brightfield morphometry, three-dimensional volumetry, and FLIM datasets were acquired from cells in fragments of multiple thalli that were dissected from the same collection. Because thallus identity was not recorded for individual cells during dissection and mounting, the cells are conservatively treated as technical rather than biological replicates throughout, and the statistical analysis is structured accordingly (see ‘Statistical analysis’). Prior to staining, samples were washed five times with distilled water (dH_2_O) to remove residual fixative and then dissected into smaller branches. The dissected tissue was then transferred to UV-resistant microcentrifuge tubes containing dH_2_O and stained overnight at 4°C with Hoechst 33342 (5 μg ml^−1^; Thermo Scientific, cat. no. 62249), supplemented with Tween 20 (0.05% v/v; Sigma-Aldrich) as a permeabilisation agent. The stained tissue was then washed and mounted in 35 mm glass-bottomed microscopy dishes (MatTek) with #1.5 coverslips, immediately before imaging (Fig. 1C).

### Confocal and FLIM imaging

Imaging was performed using an Abberior Facility Line stimulated-emission-depletion (STED) system with FLIM (Abberior Instruments GmbH, Göttingen, Germany), equipped with a multi-detector time-gated FLIM system (MATRIX), and on a Nikon spinning-disk system fitted with a Cicero confocal spinning-disk unit and an Extended Focus device (Hu et al., 2020). Morphological surveys and Hoechst nuclear imaging were carried out in point-scanning confocal mode using the STED system and a 20×/0.8 NA air objective to obtain low-magnification tissue overviews and a 60×/1.30 NA silicone-immersion objective (UPLSAPO60XS2; Olympus; pinhole 0.93 Airy units) for single-cell imaging. FLIM data were acquired using pulsed excitation and time-gated detection across three spectrally distinct emission channels: green (λem 498–551 nm), orange (λem 571–630 nm) and far red (λem 650–760 nm). Phasor-plot analysis was used to derive the mean fluorescence lifetime (τ) for each channel individually, and per-cell values were obtained from the phasor centre of mass of the cell mask. The lifetime scale of the instrument was validated against a reference fluorophore and a commercial test slide under the acquisition settings used for all *Hydrurus* FLIM data (40 MHz repetition rate, 100 ps per time bin, read from the acquisition metadata; 60×, pinhole 1.0 AU). A mono-exponential tail fit to the pooled time-correlated single-photon counting (TCSPC) decay of fluorescein in aqueous solution (3.1×10^6^ photons) recovered τ=3.93±0.01 ns against a nominal value of ≈4.0 ns, with a per-pixel median from the instrument's own fit of 4.00 ns; an Abberior CELLS four-colour test slide (cat. no. IG4COLOR-4005) imaged in the same three emission channels used here gave mono-exponential lifetimes of 2.21 ns (STAR GREEN), 2.29 ns (STAR ORANGE) and 3.01 ns (STAR RED) (Fig. S1).

The spinning-disk dataset was acquired using a Nikon ECLIPSE Ji microscope equipped with a CrestOptics CICERO spinning-disk confocal unit and controlled via NIS-Elements software.

### Image processing and quantitative morphometry

Raw Abberior data files were batch converted to Open Microscopy OME-TIFF format using a custom Python script (Ø. Ø. Fougner, University of Oslo). Nikon .nd2 image files were read in Python using the nd2 reader and processed with scikit-image, SciPy and pandas. Two imaging datasets underlie the morphometry (only the brightfield field is shown, in Fig. 2A–C): a 20× brightfield field of view (0.313 μm pixel size) for cell-shape context, and a 60× silicone-immersion, extended-depth-of-focus, stitched two-channel confocal mosaic (12,890×9871 px; 0.1127 μm pixel size). The mosaic comprises an AF647-range autofluorescence channel, used to outline the cell body, and a nuclear (Hoechst 33342) channel, used to label the nucleus; this channel is labelled ‘DAPI’ in the instrument software and consequently in the deposited file metadata, but the fluorophore used was Hoechst 33342 throughout.

Zone assignment was performed on the confocal mosaic using an explicit, reproducible rule rather than by eye. The thallus footprint was obtained from the autofluorescence channel (Gaussian smoothing, σ=2 px on 8×-decimated data; Otsu threshold; removal of objects <300 px; morphological closing with a disk of radius 6 px; hole filling; largest connected component). Two maps were then computed within this footprint: (i) radial depth, as the Euclidean distance transform of the footprint converted to micrometres, and (ii) a nuclear-density map, obtained by detecting local Hoechst maxima (minimum separation 2 px on the decimated grid; intensity threshold at the 80th percentile of in-thallus Hoechst signal) and smoothing the resulting point map with a Gaussian kernel (σ=12 px). The cores were defined as pixels with radial depth >35 μm and nuclear density above the 66th percentile of the in-thallus density distribution; margin as pixels with radial depth <18 μm or nuclear density below the 33rd percentile; and mid as the remainder. Fifteen 150 μm×150 μm ROIs (150 μm on a side, 1332×1332 px at full resolution) were then placed on a half-overlapping grid, retained only where they were ≥40% filled by thallus, assigned to the zone occupying the majority of their area, and selected by farthest-point sampling to maximise spatial spread (six margin, five mid and four core ROIs), yielding 2422 segmented cells (925 margin, 691 mid and 806 core cells).

Cell bodies were segmented from the autofluorescence channel using Cellpose-SAM (Cellpose v4 generalist model; CPU inference; flow_threshold=0.4, cellprob_threshold=0.0; automatic diameter estimation). Because aspect ratio and orientation are scale invariant, segmentation was performed on 3×-downsampled tiles (effective pixel size 0.338 μm); the resulting integer label masks were mapped back to full resolution using nearest-neighbour (order-zero) resampling, which preserves the discrete label values, and all size measurements were made on these full-resolution masks. Segmented objects smaller than 3 μm^2^ were discarded; in the brightfield data, debris was additionally excluded by area (8–400 μm^2^) and solidity (≥0.85). For each cell, area, major and minor axis lengths, aspect ratio, eccentricity, solidity and major-axis orientation were computed from the segmentation labels by second-moment (principal-component) fitting. Nuclei were localised within each cell mask as the brightest compact Hoechst region – the largest connected component of the top 20% of Hoechst intensity inside the cell – measured in the full-resolution Hoechst channel; this bright-spot approach was necessary because the Hoechst stain produced a dim diffuse whole-cell background in addition to a discrete nuclear body, so thresholding the entire channel detects the background rather than the nucleus. Cell alignment was expressed as the angular deviation of each cell's major axis from the thallus long axis (122.5°, derived from the binarised thallus skeleton) and summarised per zone by the nematic order parameter S=〈cos 2φ〉. Nuclear polarisation was quantified as the nucleus-to-cell-centroid displacement vector, expressed both as a fraction of the cell half-length and as a signed projection onto the thallus long axis. All pixel-to-micrometre conversions used the objective-specific calibrations mentioned above. Intensity displays used per-image percentile contrast stretching for visualisation only; all measurements were made on raw pixel values. Segmentation accuracy was validated against blind manual annotation: three fields of the analysed mosaic spanning all thallus zones (259 cells) were outlined manually without reference to the automated masks, and the Cellpose-SAM output scored against them by Hungarian matching on intersection-over-union (IoU). Object-level precision was 0.83, recall 0.91 and F1=0.87 at IoU≥0.5, with a median matched-cell IoU of 0.77 (Dice 0.88); errors concentrate as merges and splits of touching elongated cells in the densest strand tissue (field-wise F1 0.80–0.95) and are unbiased with respect to position along the thallus (Fig. S2).

### Three-dimensional cell volumetry

The cell volumes were measured using three-dimensional z-stacks acquired on the Abberior system with its iMSPECTOR control software and a 60×/1.30 NA silicone-immersion objective (UPLSAPO60XS2; Olympus). Each cell was recorded as a separate stack and stored as an object binary file. Only the far-red channel after maximum-likelihood-estimation deconvolution (STAR RED) was analysed; the corresponding raw, transmitted-light and Hoechst stacks were not used. Voxel calibrations were read from the embedded OME metadata (z=0.250 μm; xy=0.065 or 0.100 μm, depending on the per-cell crop), and each cell's volume was computed using its own voxel size. For segmentation, each deconvolved stack was Gaussian-smoothed (σ=1 voxel) and thresholded using Otsu's method on the non-zero voxels; the resulting mask was morphologically closed (ball filter, radius 2 voxels), reduced to its largest three-dimensional connected component, and had interior holes (vacuoles) filled in both three dimensions and slice by slice. Cell volume was calculated by multiplying the number of masked voxels by the voxel volume; surface area was obtained by marching-cube estimation, sphericity from the volume and surface area, and in-plane length, width and elongation from the maximum-intensity projection. The surface-to-volume ratio was computed per cell. For the subset of stacks containing a per-cell Hoechst z-stack (13 elongated, two round cells), the nucleus was segmented in three dimensions as the largest connected component above the 95th percentile of in-cell Hoechst intensity after Gaussian smoothing (σ=1 voxel), within a cell region defined by the far-red channel; nuclear volume, equivalent diameter and the centroid offset along the cell's in-plane major axis were computed per cell and are listed in Table S1 (the median offset changed by <0.1 μm when the threshold was varied between the 90th and 95th percentiles).

All 65 available deconvolved far-red stacks were segmented and exported. Cells from the main acquisition batch (25 elongated, 25 round) showed the far-red label filling the entire cytoplasm and were segmented cleanly (two-dimensional convex solidity 0.88–0.99). Cells from a separate exploratory acquisition (13 elongated, two round) instead showed the label confined to a thin, incomplete cortical shell with an empty interior (solidity 0.63–0.94); because these shells are open along the z-axis, intensity thresholding systematically underestimates their volume, and these 15 cells were therefore excluded from the volume comparison, although they were retained and flagged in the exported manifests. Border and z-clip checks on the remaining cells revealed no instances of clipping (one flagged cell made contact with the edge of the field of view in 37 voxels, equivalent to approximately 0.03% of its volume, and was retained), resulting in a total of 50 quality-controlled cells (25 per morphological class) being available for analysis.

### Statistical analysis

All observations were collected at a single time point, and the statistical analysis was structured accordingly. Group differences were assessed using two-sided Mann–Whitney *U* tests, and effect sizes quantified as the rank-biserial correlation. Orientational non-uniformity was tested using a Rayleigh test on doubled angles; directional bias in nuclear displacement was tested with a two-sided binomial test against a null fraction of 0.5 and with a two-sided Wilcoxon signed-rank test on the signed projections. The bimodality of the per-cell far-red lifetimes was assessed using Hartigan's dip test and, independently, by comparing one- and two-component Gaussian mixture models using the Bayesian information criterion; mixture components were fitted using 50 random restarts. In the FLIM comparison, three round cells whose acquisitions had been flagged during recording – before any analysis – were excluded from the primary analysis based on this acquisition-quality criterion (*n*=26 round); the all-cell analysis (*n*=29) is reported as a sensitivity check. We explicitly note that this choice affects the channel medians: when the flagged cells are included in the analysis, the far-red comparison is not significant (Holm-corrected *P*=0.20), but when they are excluded, the comparison is significant (*P*=0.036); the round-cell far-red bimodality remains robust when the flagged cells are excluded (dip test *P*=6.4×10^−3^; ΔBIC=49.0). Mann–Whitney *P*-values for the three emission channels were Holm corrected across channels. Because the mosaic cells originate from a single thallus and thallus identity was not recorded for individual cells in the other datasets, cell-level inferential statistics would overstate precision; for nuclear polarisation, inference used the imaging ROI as the unit of replication (*n*=15; binomial and Wilcoxon tests on ROI means), and cell-level summaries are reported as descriptive statistics only. Holm correction was likewise applied across the morphometric comparisons; the core-versus-margin aspect-ratio comparison did not survive correction (*P*=0.38), and no zone-wise cell-shape difference is claimed. Analyses were performed in Python using SciPy, scikit-learn, diptest and pandas. Sample sizes (numbers of cells) are given in the Results and figure legends.

All analyses are based on material collected on 16 February 2026 (whole-thallus mosaic: one intact thallus; brightfield morphometry, volumetry and FLIM: cells from multiple thalli of the same collection); this corresponds to the BioSample record of the BioImage Archive (BioStudies; Sarkans et al., 2017) under accession number S-BIAD4000 (https://doi.org/10.6019/S-BIAD4000).

### Use of AI-based tools

Cell segmentation was performed using Cellpose-SAM (Cellpose v4), a deep-learning segmentation model. All quantitative analysis pipelines were implemented in Python (scikit-image, SciPy, scikit-learn, pandas). Generative AI tools were used to edit the manuscript text and assemble the figures; all analyses, measurements and conclusions were performed and verified by the authors.

## Supplementary Material



10.1242/biolopen.062873_sup1 Supplementary information
