# Supplementary material for: Morphology and autofluorescence of the thallus of the golden-brown alga Hydrurus foetidus by optical and fluorescence lifetime imaging
Source: Biol Open. 2026 Oct 30;15(11):bio062873. doi: 10.1242/bio.062873 (PMC13750392; doi:10.1242/bio.062873)
Supplement: Supplementary file 1 [file biolopen-15-062873-s1.pdf]

Supplementary Figure S1 — FLIM instrument validation: reference fluorophore and 4-colour test slide

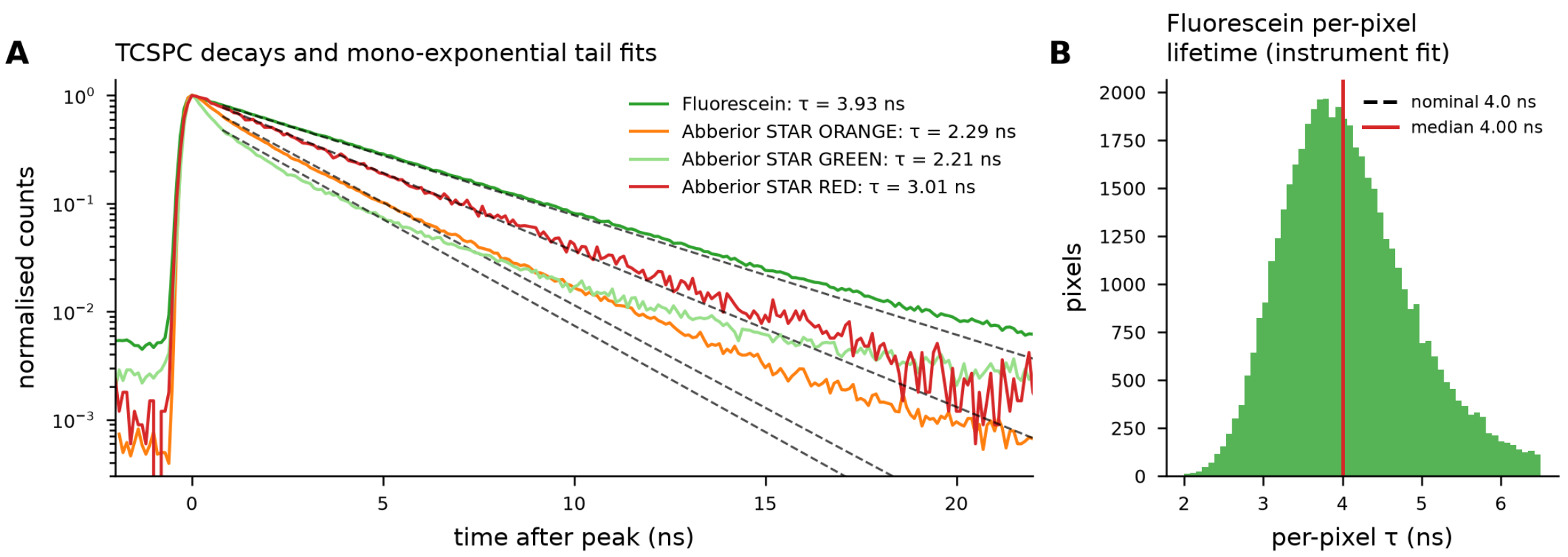

C Acquisition settings and recovered lifetimes

Sample	Channel (λem)	Measured τ (tail fit)	Per-pixel median	Reference
Fluorescein, aqueous solution	green (498–608 nm)	3.93 ± 0.01 ns	4.00 ns	4.0 ns (lit., pH ≥ 8)
Abberior CELLS 4-colour slide:				
STAR GREEN (phalloidin)	green (498–551 nm)	2.21 ± 0.02 ns	—	conjugate in cells
STAR ORANGE (Golgi)	orange (571–630 nm)	2.29 ± 0.01 ns	—	conjugate in cells
STAR RED (NPC)	far-red (650–760 nm)	3.01 ± 0.01 ns	—	3.4 ns (free dye, PBS)

Fig. S1. FLIM instrument validation with a reference fluorophore and a four-colour test slide. The fluorescence-lifetime scale of the FLIM system (Abberior Facility Line) was validated under the acquisition settings used for all *Hydrurus* FLIM data in this study: 40 MHz pulsed excitation and TCSPC histogramming at 100 ps per time bin over a 25 ns window (read from the acquisition metadata), 60× objective, pinhole 1.0 AU. (A) Pooled TCSPC decay curves (normalised to peak; logarithmic scale) for fluorescein in aqueous solution, recorded in the green channel (λex 488 nm; 3.1×10^6 photons), and for the three dyes of a commercial test slide (Abberior CELLS 4 color): STAR GREEN–phalloidin (λex 488 nm, λem 498–551 nm), STAR ORANGE–Golgi marker (λex 561 nm, λem 571–630 nm) and STAR RED–nuclear pore complex marker (λex 640 nm, λem 650–760 nm). These are the same three emission windows used for the *H. foetidus* autofluorescence analysis. Dashed lines show mono-exponential tail fits (fit window beginning 0.8 ns after the decay peak): fluorescein, τ = 3.93 ± 0.01 ns (nominal ≈ 4.0 ns in aqueous solution at pH ≥ 8); STAR GREEN, 2.21 ± 0.02 ns; STAR ORANGE, 2.29 ± 0.01 ns; STAR RED, 3.01 ± 0.01 ns (fit ± s.e.). (B) Distribution of per-pixel fluorescein lifetimes from the instrument's phasor-based fit of the same acquisition; the median (4.00 ns, red line) lies within 2% of the nominal value (dashed line). (C) Samples, emission channels and recovered lifetimes. Lifetimes of dye–protein conjugates in mounted cells are expected to differ from free-dye values; the test-slide measurements therefore demonstrate channel-to-channel consistency of the lifetime scale rather than recovery of free-dye reference values.

Biology Open • Supplementary information

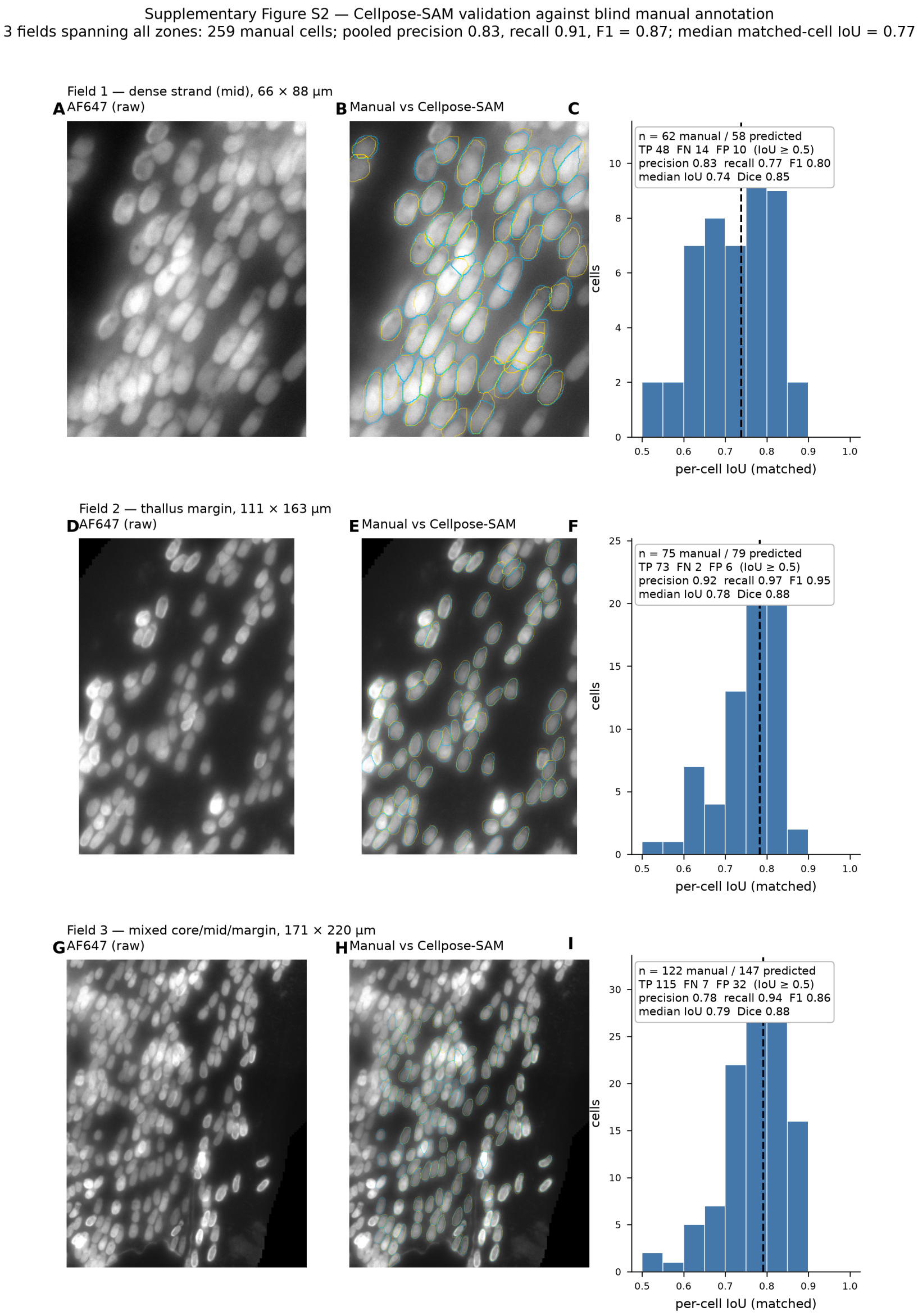

Fig. S2. Validation of automated cell segmentation against blind manual annotation.
Cellpose-SAM segmentation (Cellpose v4 generalist model, with the settings used in the main analysis: autofluorescence channel, 3× downsampling, flow threshold 0.4, cell-probability threshold 0.0) was scored against manual annotation of three fields of the analysed 60× confocal mosaic that together span all thallus zones: a dense-strand region in the mid zone (66 × 88 µm; A–C), a region at the thallus margin (111 × 163 µm; D–F) and a mixed core/mid/margin region (171 × 220 µm; G–I). Cells were outlined by hand in Fiji from the raw image without reference to the automated masks (259 cells in total; in the third field, within an annotated sub-region); cells touching the field border were excluded from both annotation and prediction. Manual and predicted objects were matched by Hungarian assignment on intersection-over-union (IoU). (A,D,G) Raw AF647 autofluorescence. (B,E,H) Manual outlines (yellow) overlaid with Cellpose-SAM outlines (cyan; coincident boundaries appear green). (C,F,I) Per-cell IoU of matched objects (dashed line, median), with object-level detection scores at a match threshold of IoU ≥ 0.5: dense strand, precision 0.83, recall 0.77, F1 0.80 (n = 62 manual cells); margin, 0.92, 0.97 and 0.95 (n = 75); mixed field, 0.78, 0.94 and 0.86 (n = 122). Pooled over all fields: precision 0.83, recall 0.91, F1 0.87; median IoU of matched cells 0.77. Residual errors are concentrated in the densest tissue, as merges and splits of touching elongated cells, and are unbiased with respect to position along the thallus.

Table S1. Three-dimensional nuclear measurements from the per-cell Hoechst z-stacks. Nuclei were segmented in 3D for the subset of volumetry acquisitions that include a per-cell Hoechst (DAPI-channel) z-stack (13 elongated, 2 round cells; acquisition batch excluded from the volume comparison because of its cortical far-red labelling, which does not affect the nuclear channel). Nucleus: largest connected component above the 95th percentile of in-cell Hoechst intensity after Gaussian smoothing ($\sigma = 1$ voxel), within a cell region defined from the far-red channel. Offset is the distance between the nuclear and cell centroids projected onto the cell's in-plane major axis; fractional offset is expressed relative to the cell half-length. Elongated cells: median nuclear equivalent diameter 2.47 µm, median offset 1.80 µm (fractional offset 0.41). The two round cells are reported descriptively and support no morphotype comparison. The median offset changed by < 0.1 µm when the segmentation threshold was varied between the 90th and 95th percentiles.

**Cell (acquisition group)**	**Morphotype**	**Nuclear volume (µm^3)**	**Nuclear equivalent diameter (µm)**	**Nuclear offset along major axis (µm)**	**Fractional offset (of half-length)**	**Cell in-plane length (µm)**
Overview 3/Cell2/10:36:23	elongated	7.2	2.39	1.80	0.38	9.4
Overview 3/Cell3/10:38:29	elongated	7.9	2.47	2.66	0.52	10.2
Overview 3/Cell4/10:41:01	elongated	6.1	2.26	2.22	0.58	7.6
Overview 3/Cell1/10:06:00	elongated	5.5	2.19	1.55	0.35	8.9
Overview 3/Cell2/10:09:08	elongated	3.7	1.91	1.67	0.42	8.0
Overview 3/Cell3/10:11:10	elongated	8.9	2.57	1.27	0.29	8.9
Overview 3/Cell4/10:13:18	elongated	5.6	2.20	1.55	0.31	9.9
Overview 2/cell1/11:40:04	elongated	9.5	2.63	2.56	0.55	9.2
Overview 1/Cell1/10:56:42	elongated	8.4	2.52	2.94	0.58	10.1
Overview 1/Cell2/10:59:34	elongated	8.1	2.49	1.23	0.26	9.6
Overview 1/Cell3/11:01:34	elongated	9.8	2.66	2.53	0.41	12.3
Overview 1/Cell4/11:04:16	elongated	6.9	2.36	2.26	0.52	8.7

Biology Open • Supplementary information

Overview 1/Cell5/11:08:11	elongated	13.7	2.97	0.64	0.12	10.4
Overview 3/Cell1/10:34:26	round	3.5	1.88	3.00	0.82	7.3
Overview 3/Cell5/10:15:58	round	8.2	2.50	0.60	0.13	9.1

Biology Open • Supplementary information
