# Supplementary material for: Morphology and autofluorescence of the thallus of the golden-brown alga Hydrurus foetidus by optical and fluorescence lifetime imaging
Source: Biol Open. 2026 Oct 30;15(11):bio062873. doi: 10.1242/bio.062873 (PMC13750392; doi:10.1242/bio.062873)
Supplement: Supplementary file 2 [file biolopen-15-062873_review_history.pdf]

Morphology and autofluorescence of the thallus of the golden-brown alga Hydrurus foetidus by optical and fluorescence-lifetime imaging

Jens Ådne Rekkedal Haga, Live Lislerud Vestli, Kjetill Sigurd Jakobsen, Julien Alain Andre Resseguier, Felix Margadant, Oddmund Bakke and Xian Hu
10.1242/bio.062873

Editor: Catherine L. Jackson

Review timeline
Original submission: 12 August 2026
Editorial decision: 20 August 2026
First revision received: 27 August 2026
Editorial decision: 7 September 2026
Second revision received: 27 September 2026
Accepted: 30 September 2026

Original submission

First decision letter

MS ID#: bio.062873

MS Title: Morphology and autofluorescence of the golden-brown alga Hydrurus foetidus by optical and fluorescence-lifetime imaging

Authors: Xian Hu, Jens Ådne Rekkedal Haga, Live Lislerud Vestli, Felix Margadant and Oddmund Bakke

I have now reached a decision on the above manuscript.

The reviewer reports are shown at the bottom of this email.

As you will see, the reviewers raised a number of substantial criticisms that prevent me from accepting the paper at this stage.

They suggest, however, that a revised version might prove acceptable, if you can address their concerns. If you think that you can deal satisfactorily with the criticisms on revision, I would be pleased to see a revised manuscript. We would then return it to the reviewers.

At this stage, we also ask you to ensure your manuscript complies with our formatting guidelines. Provided you are able to fully address the referees' comments, we are positive about publication of your paper (we accept over 95% of revision submissions) and therefore hope you won't mind any extra work involved in reformatting your manuscript at this point.

Please upload both a 'clean' version of your Word file, along with a highlighted version clearly showing where you have made changes in the revised manuscript. Please avoid using 'Track changes' in Word files as these are lost in PDF conversion.

I should be grateful if you would also provide a point-by-point response detailing how you have dealt with the points raised by the reviewers in the 'Response to Reviewers' box. Please attend to

© 2026. Published by The Company of Biologists under the terms of the Creative Commons Attribution License (https://creativecommons.org/licenses/by/4.0/).

all of the reviewers' comments. If you do not agree with any of their criticisms or suggestions please explain clearly why this is so.

Reviewer 1

Comments for the author

General comment
The manuscript presents a novel and interesting methodology and provides a valuable baseline characterization of H. foetidus. The combination of morphometric analyses with autofluorescence and FLIM imaging is well suited to the aims of a Methods & Techniques article, and the image analysis workflow is described in considerable detail. I found the visual material generally clear and useful. However, this may be related to the version provided for review, but in some figures the text appears rather small and difficult to read. If possible, increasing the font size in these figures would improve their readability.

Specific comments

- It would be helpful to clarify how many independent biological replicates were included in the morphometric analysis. In particular, were the segmented cells obtained from several or from a small number of individuals? This information would help readers better understand how the statistical comparisons should be interpreted.
- Samples were fixed with paraformaldehyde prior to imaging. Since fixation may affect autofluorescence signals, could the authors briefly comment on whether this was considered when interpreting the results, either in the Methods or Discussion?
- Could the authors clarify whether any form of system validation or reference measurement was performed? For example, the use of a sample with known or expected behaviour could help readers assess the reliability and reproducibility of the reported fluorescence lifetime values.
- Was the accuracy of the cell segmentation checked against manual annotation, even for a subset of cells, given that the model used was not specifically trained on H. foetidus?
- Could the authors clarify why the three cell acquisitions flagged during the analysis were retained in the main FLIM statistical analysis? The manuscript mentions that excluding these observations changes one of the reported p-values. It may also be useful to present the analysis excluding these observations as a sensitivity check.
- Regarding the statistical tests that were performed, could the authors comment on whether corrections for multiple comparisons were considered or applied?
- The results are novel and interesting, but the Discussion could be strengthened by placing the autofluorescence and FLIM findings in a broader context. At present, these results are only briefly discussed in relation to studies of other organisms. It would be useful to include more comparisons with related studies in other algae and photosynthetic organisms, discussing how the present findings compare with previous observations. This would help provide a more complete interpretation of the results and the technique.

Reviewer 2

Comments for the author

All my comments and suggestions for improvements are attached in the relevant document.

I am briefly stating here the reasons for the "No".

- Biological replicates. If the sampling if from one season, one habitat, one sampling time, counting cells fromt he same sample is not biological repeat but rather technical. I would expect that more thalli used to have biological repeats. Fewer cells per thallus would still strnegthen reproducibility adequately.
- Raw data: I guess there are files of raw data (counts, images etc), and they should be available.
- Title: Does Hydrurus foetidus body has only thallus? The work is focused on the thallus. Maybe the title should not be so general but mention the thallus

© 2026. Published by The Company of Biologists under the terms of the Creative Commons Attribution License (https://creativecommons.org/licenses/by/4.0/).

All my comments and suggestions for improvements are available in a separate marked up pdf file of the manuscript.

The Manuscript titled "*Morphology and autofluorescence of the golden-brown alga Hydrurus foetidus by optical and fluorescence-lifetime imaging*" by Xian Hu; Jens Ådne Rekkedal Hagal; Live Lislerud Vestli; Felix Margadant; Oddmund Bakke, is well writen and with the required detailed description of biological resources of the chrysophyte and methods, such as FLIM with phasor plot analysis and Cellpose-SAM. Besides the description of their findings, authors draw a number of conclusions and hypotheses, some of which remain to be tested.

Methodology

- In the introduction, authors mention that the chrysophyte is maintained in a laboratory culture. Then in the "Sample collection and preparation" paragraph the sampling of the chrysophyte in its natural habitat (plateau) is explained. I can guess that the experiments performed are on the latter sample, but this should be mentioned. In either case of sampling, the experiments are performed on rather technical repeats of the same biological sample. Therefore, statistical analysis (description and explanation) should be adapted accordingly.
- In the case of the sample from a laboratory culture, the conditions are explicitly laboratorial and exclude natural. In the case of the sample from the plateau, the conditions are natural but momentary. This could impose seasonal, life-cycle-stage, or population effects on the morphological and developmental observations. Therefore, expressing conclusions and hypotheses should be adapted to the growth conditions of the samples used.
I'm not suggesting that same experiments should be done in different conditions for the current manuscript so more precise and inclusive conclusions could be drawn. But this could be included for future experimental approach.
- It is not clear and not commented how reliable cell volumetric measurements can be in fixed samples. Especially when analyzing cells with variable differentiation, shape and *in situ* location. Maybe authors can comment on this by including a possible effect by fixation or maybe a testing that fixation effects are minor/negligible or at least uniform for all cells.

Conclusions and Hypotheses

- Differentiation state of round vs elongated cells (page 6, line 34 and onward)

There are a handful of approaches to explore this. EdU staining for instance could distinguish actively dividing or differentiated cells. This method could also access the differences between samples from plateau and lab cultures. Maybe simple PI staining would also access the level of live to dying cells.

- **FLIM-based characterization of chlorophyll photophysical state (page 10, line 4 and onward)**

This interpretation of the single-cell phasor results attributed to the photophysical state of the chlorophyl could be easily tested by same FLIM experiments upon PSII electron transport blocker (DCMU).

- **Cytoskeleton-assisted nuclear anchoring (page 7, line 53 and onward)**

This hypothesis can easily get tested with anti-tubulin immunolabeling or live SiR tubulin labeling. This would allow the claims to be rather substantiated than just supported by citations.

Testing these any of these conclusions or hypotheses would elevate the impact and output of the manuscript, however simple commenting as future approaches shouldn't prevent publishing it.

Formatting

- Same Figures are not correctly mentioned in text. For instance, Figure 2 in page 12, line 53. It is not clear which part of the figure is commented.
- It is not clear to me is some parts of the Figures are commented in the text. It seems like figures are there without being commented in the text. For example, Figures 2F to K, 3E
- There are some problems with the numbering and formatting of figures. The number of the figure is not right above the actual figure and this is confusing. Then the font size in most figures varies and some are very small
- Phasor plot background fill is black and the individual points are not easily discerned. Dark gray in darker gray or black, should not be the case.

© 2026. Published by The Company of Biologists under the terms of the Creative Commons Attribution License (https://creativecommons.org/licenses/by/4.0/).

- Lastly the conclusions and outlook paragraph could be enriched with the connection of the hypotheses mentioned through the text and future approaches to test them.
- There are several sentences which are long, complicated and tough to comprehend,

Reviewer's Responses to Questions

Experimental quality
Are experiments performed using appropriate methods that will answer the question (or test the hypothesis or support the observations) posed by the authors? Is the right tool used for the job?
If 'No', please indicate reasons in Comments for Author box below.
Reviewer #1:
- Yes

Reviewer #2:
- Yes

Were the data analyzed using appropriate statistical tests?
If 'No', please indicate reasons in Comments for Author box below.
Reviewer #1:
- Yes

Reviewer #2:
- Yes

Reproducibility
Were experiments performed using adequate number of biological replicates?
If 'No', please indicate reasons in Comments for Author box below.
Reviewer #1:
- No

Reviewer #2:
- No

Is there sufficient raw data to assess the rigor of the analysis?
If 'No', please indicate reasons in Comments for Author box below.
Reviewer #1:
- Yes

Reviewer #2:
- No

Does the methods section provide sufficient detail to permit reproducibility?
If 'No', please indicate reasons in Comments for Author box below.
Reviewer #1:
- Yes

Reviewer #2:
- Yes

Completeness
Are the manuscript's conclusions supported by the data?
If 'No', please indicate reasons in Comments for Author box below.
Reviewer #1:
- Yes

© 2026. Published by The Company of Biologists under the terms of the Creative Commons Attribution License (https://creativecommons.org/licenses/by/4.0/).

Reviewer #2:
- Yes

Scholarship
Do the authors cite and discuss the merits of data that would argue for and against their conclusion?
If 'No', please indicate reasons in Comments for Author box below.
Reviewer #1:
- No

Reviewer #2:
- Yes

Does the manuscript title & abstract accurately reflect the contents of the manuscript, without hyperbole?
If 'No', please indicate reasons in Comments for Author box below.
Reviewer #1:
- Yes

Reviewer #2:
- No

First revision

Author response to reviewers' comments

We thank both reviewers for detailed and constructive reports. Three points deserve to be said at the outset.

First, we accept the central criticism, and we have also clarified the sample provenance, which the submitted manuscript left ambiguous. All material derives from a single collection on one date. The whole-thallus spatial analyses (Fig. 3) come from one intact thallus, selected as the most representative of many thalli examined, for imaging-feasibility reasons we explain under point 1.2. The brightfield morphometry, volumetry and FLIM datasets sampled cells from dissected fragments of multiple thalli of that collection; however, because thallus identity was not recorded for individual cells during dissection and mounting, these cells cannot be assigned to individuals retrospectively. We have therefore conservatively restructured the statistical treatment so that no inferential statistic treats cells as independent biological replicates, and the wording of the conclusions has been adapted to the material actually sampled.

Second, two constraints shape several of our answers, and we state them plainly here rather than repeating them under each point. *Hydrurus foetidus* does not survive above approximately 12 °C, and the Finse Research Station has no high-resolution microscope; material must therefore be fixed on site and transported to Oslo for imaging, so experiments requiring live cells at the microscope — live SiR-tubulin labelling, paired live-versus-fixed imaging, time-lapse — are not available to us. In addition, *H. foetidus* is a cold-water winter and spring alga whose collection window at Finse is approximately November to February, so no new field material can be obtained within the revision period.

Third, we have therefore taken the course the reviewer explicitly allowed — that commenting on future approaches "shouldn't prevent publishing it". Every experimental suggestion that requires new field material (additional thalli, EdU pulse-labelling, DCMU treatment, tubulin immunolabelling) is addressed in the revision as an explicitly described future approach, designed

© 2026. Published by The Company of Biologists under the terms of the Creative Commons Attribution License (https://creativecommons.org/licenses/by/4.0/).

around the field constraints above, rather than as new data. What we have added within this revision is everything achievable from existing material and instrumentation: a full statistical reanalysis with corrected units of replication and multiple-comparison control, a new cross-channel analysis that strengthens the FLIM interpretation from internal evidence, three-dimensional nuclear measurements recovered from the deposited volumetry stacks, an instrument validation against a lifetime standard, and a validation of the automated segmentation against manual annotation (Supplementary Fig. 2).

Reviewer 1

1.1 Figure fonts are too small; unify with Fig. 3A

All figures have been rebuilt on a single three-level type scale, with panel titles, axis labels and tick/legend text at consistent sizes across every figure including Fig. 3A.

1.2 How many independent biological replicates?

For the whole-thallus spatial analyses (Fig. 3): one. For the cell-level datasets (brightfield morphometry, volumetry, FLIM): cells were sampled from dissected fragments of multiple thalli, all collected next to the Finse Research Station on 17 February 2026 — but thallus identity was not recorded for individual cells during dissection and mounting, so cells cannot be assigned to individuals retrospectively, and we conservatively treat all cell-level data as technical rather than biological replicates. The Methods now state this provenance explicitly rather than leaving it to be inferred, and we have removed any phrasing that implied independent replication.

We wish to explain how this specimen was chosen, because it was not arbitrary. Many thalli were examined under the same microscope, and the imaged specimen was selected as the most representative of them. Capturing a tissue-wide image of this organism is difficult with our preparation: the tissue is large and optically clouded, and most thalli are too big to fit into a single z-stack of practical size — a whole-thallus acquisition should stay within roughly 25 GB per stack and a z-range below 100 µm. The imaged thallus was the only specimen in which the entire structure could be captured intact within these limits. Whole-thallus quantitative imaging was therefore performed on this specimen, while the others informed our qualitative judgement that its architecture is typical. We are additionally uploading single-field-of-view z-stacks from further thalli to the BioImage Archive open-access deposition (S-BIAD4000: https://doi.org/10.6019/S-BIAD4000), so that other researchers have access to raw data beyond the analysed specimen. We accept that neither the qualitative survey nor these additional stacks substitute for formal biological replication, which is why the statistical treatment and the conclusions have been restructured as described here.

Because the collection window for this cold-water winter and spring alga is approximately November to February, no additional thalli can be obtained within the revision period. We have therefore addressed the point in the two ways available to us. First, the statistical treatment has been restructured so that it is honest about the material: no analysis treats cells as independent biological replicates (see our response to point 1.7), and the manuscript now describes the findings as a characterisation of the sampled material rather than of the species in general. Second, the revised closing section specifies the replication study we consider necessary before generalising: five to six thalli across at least two collection dates and two stream positions, with every analysis re-run using the thallus as the unit of replication. A power analysis on the between-region variability of the existing data indicates that three to four thalli suffice for the nuclear-polarisation test and that the volume comparison reaches adequate power at five, which is why five to six is the stated target; we note that this calculation estimates between-thallus variance from between-region variance within a single thallus and is therefore a lower bound.

1.3 Does paraformaldehyde fixation affect the autofluorescence interpretation?

This is a fair concern and we cannot address it with paired live imaging, for the logistical reasons given above. We have therefore added an explicit treatment to the Discussion resting on four arguments and one acknowledged limit.
First, the choice of fixative was made with exactly this concern in mind. Glutaraldehyde preserves ultrastructure better, but its free aldehyde groups form fluorescent Schiff-base adducts with tissue amines and generate strong, broad-spectrum autofluorescence — which is disqualifying in a study

© 2026. Published by The Company of Biologists under the terms of the Creative Commons Attribution License (https://creativecommons.org/licenses/by/4.0/).

whose readout is autofluorescence itself. Paraformaldehyde was chosen as the standard compromise for fluorescence work: adequate structural preservation at light-microscopy resolution with the least perturbation of the native fluorescence signal. We further note that PFA-induced autofluorescence, where it occurs, is concentrated in the blue-green emission range; the central result of this study lies in the far-red channel (650–760 nm), the spectral region least affected by fixative-derived signal. An additive fixative background also could not produce the observation we report — a bimodal lifetime distribution with an empty gap, confined to one morphotype — since a contaminating fluorophore of fixed lifetime would shift all cells, not split one class in two.

Second, the volumetric comparison is a ratio. All cells were fixed in a single batch under identical conditions, and the elongated-to-round median volume ratio is 1.32 (356 versus 270 μm^3, n = 25 per class). Uniform isotropic shrinkage by any factor k scales both volumes by k^3 and leaves the ratio unchanged, so the conclusion that elongated cells are larger is robust to fixation artefacts acting equally on both morphotypes.

Third, the morphotype classification rests on dimensionless quantities, which are scale-free and therefore insensitive to uniform volume change. In the 50-cell volumetric dataset, in-plane elongation separates the morphotypes strongly (medians 2.43 versus 1.07; Mann–Whitney $P = 1.4 \times 10^{-9}$), whereas sphericity is similar between them (0.61 versus 0.63; $P = 0.59$) — consistent with the volume difference arising from axial extension rather than surface elaboration. These values are now reported in the Results.

Fourth, the FLIM data themselves demonstrate that fixation preserves the photophysical contrast this paper reports: all FLIM acquisitions are from PFA-fixed cells, and they nonetheless resolve a cleanly bimodal far-red lifetime distribution with an empty gap between modes. Whatever fixation does to these cells, it does not homogenise the lifetime differences.

We state the residual limitation directly: absolute volumes may be systematically offset from live values, the ratio argument fails if shrinkage is *differential* between morphotypes, and neither can be quantified without live imaging. We cite the relevant fixation and autofluorescence literature at this point in the Discussion.

1.4 Was the system validated against a reference sample?

We have now performed this validation and report it in a new supplementary figure S1. A reference fluorophore of known lifetime — fluorescein in aqueous solution, nominal $\tau \approx 4.0$ ns — and three Abberior STAR dyes from the Abberior CELLS 4-colour test slide (IG4COLOR-4005) were measured on the same instrument, objective (60×, pinhole 1.0 AU) and acquisition settings used for the *Hydrurus* data (Supplementary Fig. 1). A mono-exponential tail fit to the pooled TCSPC decay of fluorescein (3.1×10^6 photons; time base of 100 ps per bin at 40 MHz repetition rate, read from the acquisition metadata) recovered $\tau = 3.93 \pm 0.01$ ns, and the median of the instrument's own per-pixel lifetime fit was 4.00 ns — both within 2% of the nominal value. The same tail-fit procedure applied to the three Abberior STAR dyes, imaged under the same conditions, recovered lifetimes in each of the three emission channels used in the manuscript (STAR GREEN 2.21 ns, STAR ORANGE 2.29 ns, STAR RED 3.01 ns), demonstrating that the lifetime scale is consistent across all three spectral channels of our analysis, not only the green channel in which fluorescein emits.

1.5 Segmentation accuracy was not validated against manual annotation

We have now performed this validation and report it in a new supplementary figure S2. Three fields of the analysed mosaic, together spanning all three thallus zones (dense mid-zone strand tissue, thallus margin, and a mixed core/mid/margin region), were manually annotated blind to the automated masks — 259 cells in total. Against the Cellpose-SAM output we report object-level precision 0.83, recall 0.91 and F1 = 0.87 (at IoU ≥ 0.5), with median matched-cell IoU 0.77 and Dice 0.88. Agreement is highest in the sparser margin tissue (F1 0.95) and lowest in densely packed strand tissue (F1 0.80), where the residual errors are merges and splits of touching elongated cells; boundary accuracy of matched cells is uniformly high (per-field median matched-cell IoU 0.74–0.79). These error modes are unbiased with respect to position along the thallus, so they do not affect the zone-wise comparisons in the manuscript. We agree this validation is necessary:

© 2026. Published by The Company of Biologists under the terms of the Creative Commons Attribution License (https://creativecommons.org/licenses/by/4.0/).

Cellpose-SAM is a generalist model and H. foetidus was not represented in its training data. The Methods now state this caveat where the segmentation is introduced.

1.6 Why were three acquisition-flagged cells retained in the main analysis?

On reflection we agree the retention was the wrong choice, and we have inverted it. The three cells were flagged during recording, before any analysis, on acquisition-quality grounds; that is a principled, analysis-independent exclusion criterion. The revised manuscript therefore uses the flagged-excluded dataset as the primary analysis, states the exclusion rule where it is applied, and reports the all-cell analysis as a sensitivity check.

We also correct an overstatement in the submitted Methods, which said that excluding these cells "does not alter any conclusion". That is not accurate. With the three cells retained, the far-red channel comparison is not significant (P = 0.20); with them excluded it is (P = 0.036). The exclusion therefore does change the far-red conclusion, and the revised text says so.

1.7 Was multiple-comparison correction considered?

It has now been applied. Holm correction across the three emission channels gives:

Channel	As published (n = 27 vs 29)		Flagged excluded (n = 27 vs 26)	
	P raw	*P* Holm	*P* raw	*P* Holm
Far-red	0.2036	0.2036	0.0357	0.0357
Orange	0.0687	0.1373	0.0070	0.0140
Green	0.00097	0.0029	0.000027	0.000082

Two consequences, which we distinguish carefully. Correction does not alter the far-red verdict in the as-published analysis — Holm leaves the largest of three P values unchanged, so far-red is P = 0.20 both before and after correction, as the submitted Fig. 4 legend already reported. Correction does, however, change the orange channel: P = 0.069 becomes P = 0.137, and we have removed language describing an orange-channel trend in the all-cell analysis. In the primary (flagged-excluded) analysis all three channels remain significant after correction.

Related correction to the polarisation statistics. Prompted by both reviewers' replication concern, we recognised that the nuclear-polarisation P values in the submitted manuscript (binomial and Wilcoxon, $P < 10^{-200}$, n = 2422 cells) treat individual cells from one thallus as independent, which they are not. We have recomputed with the imaging region of interest as the unit of replication: 15 of 15 regions show nuclear displacement biased to the same pole, binomial $P = 6.1 \times 10^{-5}$, Wilcoxon on region means $P = 6.1 \times 10^{-5}$, mean of region means −1.88 µm (SD 0.57). The finding is unchanged; the inflated precision is gone. Cell-level values are retained as descriptive statistics only.

We also applied Holm correction across the morphometric comparisons. The nuclear-polarisation results survive; the core-versus-margin aspect-ratio comparison does not (P = 0.38), and we have removed the corresponding claim.

1.8 The Discussion should engage data arguing for and against the conclusions

We have substantially expanded the Discussion to place the lifetime measurements in the context of the chlorophyll photophysics literature — the established sub-nanosecond to ~2 ns range for photochemically quenched, functionally coupled chlorophyll *a* versus several nanoseconds for uncoupled chlorophyll, which is the axis our 1.4 ns and 3.3 ns poles fall on; prior FLIM studies using photosynthetic inhibitors; non-photochemical-quenching lifetime shortening; and comparable algal FLIM measurements under stress. We also now cite work on tissue-level light and photosynthesis gradients in macroalgal thalli as a functional analogue to the medulla-cortex organisation we describe, and the literature on fixation-induced changes in autofluorescence in support of our response to point 1.3.

© 2026. Published by The Company of Biologists under the terms of the Creative Commons Attribution License (https://creativecommons.org/licenses/by/4.0/).

Reviewer 2

2.1 The experiments are technical repeats of one biological sample

We agree, and we address this as described under Reviewer 1.2. The statistical treatment has been restructured so that no analysis treats cells as independent biological replicates, the conclusions are worded as a characterisation of the sampled material, and the closing section specifies the multi-thallus replication study that must precede any generalisation.

2.2 Raw data must be available

The complete imaging dataset is deposited in the BioImage Archive under accession **S-BIAD4000** (DOI 10.6019/S-BIAD4000), and is cited in the Data availability statement and Methods. The deposition comprises the confocal thallus surveys, the single-cell confocal stack, the FLIM acquisitions in all three emission channels, and the volumetry z-stacks. We are additionally depositing the per-cell measurement tables (morphometry, lifetimes, volumetry) and the complete analysis code as supplementary files, so that every reported statistic can be reproduced from the tables without access to the raw images.

2.3 The title refers to the organism, but the work describes the thallus

Accepted. The title now reads "Morphology and autofluorescence of **the thallus of** the golden-brown alga *Hydrurus foetidus* by optical and fluorescence-lifetime imaging", and we have checked the abstract for the same over-generalisation.

2.4 Laboratory culture is mentioned in the Introduction but the sampling is from the plateau

We apologise for the ambiguity — the sentence was intended as a statement about the published literature, not about this study's material, and we can see that it does not read that way. No cultured material was used anywhere in this work. Every specimen examined was field-collected at Finse on 17 February 2026 and fixed on site.

We have disambiguated the Introduction sentence to make clear that the culture report belongs to previous authors, and added an explicit statement of provenance confirming that all material in this study was field-collected and fixed on site.

We have also added the sampling rationale the reviewer asked for: the study deliberately targets the organism in its natural habitat, because the psychrophilic, rheophilic growth form and the differentiated thallus architecture that motivate this work develop in fast-flowing cold streams and are not reproduced in culture.

2.5 How reliable is volumetry in fixed samples?

Addressed in full under Reviewer 1.3. In brief: the morphotype comparison is a ratio and is invariant to uniform shrinkage; the classification rests on dimensionless shape measures; the fixed-cell FLIM data show that fixation does not abolish the physiological contrast; and we state explicitly that absolute volumes may be offset and that differential shrinkage between morphotypes cannot be excluded without live imaging.

2.6 Momentary, single-season sampling limits the conclusions

Agreed, and we have adapted the wording throughout rather than claiming generality we cannot support. Observations are now presented as describing the sampled material — a February population at one site — and the closing section frames the work as a baseline characterisation whose seasonal and life-cycle generality remains to be established. We are grateful that the reviewer distinguished this from a demand for new conditions.

2.7 Differentiation state of round versus elongated cells — EdU, PI

We thank the reviewer for this suggestion and have incorporated it into the revised closing section as a specified future approach, in the form compatible with our field constraints: fragments of freshly collected thallus pulsed with EdU in stream water on site at ambient temperature, fixed at the end of the pulse, with click detection performed in Oslo alongside Hoechst counterstaining. This would score S-phase cells by morphotype and test the proposal directly. Because *H. foetidus* is psychrophilic and no EdU incorporation time is published for this organism, two pulse durations

© 2026. Published by The Company of Biologists under the terms of the Creative Commons Attribution License (https://creativecommons.org/licenses/by/4.0/).

would be run. Executing this requires the next collection window (November–February) and therefore lies outside the present revision, as the reviewer's closing remark anticipated.

We have not adopted the propidium iodide suggestion. PI discriminates live from dying cells only in cells that are alive at the moment of imaging; after on-site fixation and transport, PI labelling would not be interpretable as a viability measure.

We can also report progress on this question from data already in hand. A subset of the volumetry acquisitions includes a per-cell nuclear (Hoechst 33342) z-stack alongside the far-red channel — 13 elongated and 2 round cells, from the acquisition batch excluded from the volume comparison because of its cortical far-red labelling (an exclusion that does not affect the nuclear channel). The revision reports this first three-dimensional, fixed-material characterisation of nuclear organisation: in elongated cells the nucleus (equivalent diameter ≈ 2.5 μm) lies a median of 1.8 μm from the cell centre along the major axis (fractional offset 0.41 of the half-length), in good agreement with the mosaic-derived two-dimensional displacement (median 2.5 μm; 46% of half-cell-length). Too few round-cell nuclear stacks are available (n = 2) for a statistical morphotype comparison; these are reported descriptively in the new supplementary Table S1, which lists all per-cell nuclear measurements, and the EdU experiment described above remains the designed test of the differentiation-state question.

2.8 The chlorophyll photophysical-state interpretation could be tested with DCMU

We agree this is the decisive experiment, and the revised closing section now describes it as a designed future approach adapted to our constraints: paired fragments from the same thallus incubated in stream water with and without DCMU on site, in the dark at ambient stream temperature, fixed at matched timepoints, and imaged in Oslo under acquisition settings identical to the present FLIM data. The paired untreated fragment is essential, since without it a treatment effect could not be separated from between-thallus variation. If the interpretation is correct, closing reaction centres should lengthen the short-lived far-red component and compress the gap between the two lifetime modes. A small dose series would be required, as no DCMU concentration has been established for this organism. Like the EdU experiment, this requires fresh material from the next collection window and therefore lies outside the present revision.

In the meantime, further analysis of the existing data strengthens the interpretation using internal evidence rather than citation alone. The eleven round cells occupying the long-lifetime mode are elevated in **all three** emission channels simultaneously, not in the far-red alone (Holm-corrected $P = 3.7 \times 10^{-5}$ for each channel). The channels co-vary strongly across round cells (far-red versus orange Spearman $\rho = 0.82$, $P = 5.6 \times 10^{-8}$; orange versus green $\rho = 0.97$, $P = 8.7 \times 10^{-18}$), and the fold-change is graded by wavelength (far-red 2.3×, orange 1.8×, green 1.3×). The high-lifetime state is confined to round cells: none of 27 elongated cells exceeds 3.0 ns in the far-red, versus 11 of 29 round cells.

A single-channel instrumental artefact — detector behaviour, photon statistics, focus — would not produce a wavelength-graded, cross-channel-coherent shift restricted to one morphotype. These observations are consistent with a whole-cell physiological state difference, which is what the DCMU experiment will test directly.

We have also verified that the bimodality does not depend on the three acquisition-flagged cells discussed under Reviewer 1.6: the long-lifetime mode contains eleven cells, of which eight are unflagged and span 3.03–3.55 ns, so the mode persists on exclusion; no round cell falls in the 1.66–3.03 ns interval. Hartigan's dip test continues to reject unimodality after exclusion ($P = 6.4 \times 10^{-3}$), and the two-component mixture remains strongly preferred (ΔBIC +49.0).

2.9 Cytoskeleton-assisted nuclear anchoring — anti-tubulin or live SiR-tubulin labelling

Of the two approaches the reviewer offers, anti-tubulin immunolabelling is the one compatible with our material, since live SiR-tubulin labelling would require living cells at the microscope. The revised closing section now names it as the designated test of this hypothesis, applicable to fixed tissue from a future collection. We also flag one genuine risk there: tubulin epitope conservation in chrysophytes is not established, so antibody cross-reactivity may fail. In the revised text the nuclear-anchoring proposal is presented explicitly as a hypothesis supported by the literature cited, not as a substantiated claim, and the wording has been checked to that standard.

© 2026. Published by The Company of Biologists under the terms of the Creative Commons Attribution License (https://creativecommons.org/licenses/by/4.0/).

2.10 Figures 2F-K and 3E are not commented on in the text
We have audited every panel against the text. Panels that carry an argument are now discussed explicitly; those that do not have been removed, which also brings the display-item count within the journal limit.

2.11 Figure numbers are not adjacent to their figures; font sizes vary and some are very small
The adjacency problem was an artefact of the submitted layout: figure numbers and legends now sit with their figures, and each figure is supplied as a separate file. The font-size problem is addressed by the rebuild described under Reviewer 1.1 — all figures now share a single three-level type scale, so no panel falls below the minimum legible size.

2.12 The dark phasor-plot background obscures individual points
The phasor plots have been redrawn on a light background with dark markers, and the previously invisible axis is now rendered.

2.13 Sentences are too long (pages 8 and 9)
The two sentences flagged have been split, and we have made a pass through the manuscript for others of the same kind.

2.14 The Conclusions do not connect the hypotheses to ways of testing them
The closing section has been rewritten so that each hypothesis is paired explicitly with the experiment that would test it — EdU pulse-labelling for the differentiation-state proposal, DCMU treatment for the chlorophyll photophysical-state interpretation, and tubulin immunolabelling for cytoskeletal nuclear anchoring — together with the material constraints that determine when each becomes feasible.

2.15 Ambiguous figure citation, page 12 line 53
Corrected. Figure callouts throughout now name specific panels rather than whole figures.

Summary of changes
1. Title amended to specify the thallus.
2. Biological-replicate status stated explicitly; conclusions reworded as a characterisation of the sampled material; the multi-thallus replication study specified in the closing section.
3. Nuclear-polarisation statistics recomputed with the imaging region as the unit of replication, replacing cell-level P values.
4. FLIM analysis restructured with the acquisition-flagged exclusion as primary; Holm correction applied across channels; the "does not alter any conclusion" statement corrected.
5. Core-versus-margin aspect-ratio claim withdrawn after correction.
6. New analysis establishing that the lifetime shift is coherent across all three emission channels and confined to round cells.
7. First three-dimensional characterisation of nuclear position added from the per-cell Hoechst z-stacks (13 elongated, 2 round cells), confirming the mosaic-derived apical nuclear offset in elongated cells; per-cell values in the new supplementary Table S1.
8. Fixation effects on volumetry addressed by ratio invariance, dimensionless shape measures and the fixed-cell FLIM contrast, with limits stated.
9. Introduction disambiguated regarding laboratory culture; provenance and sampling rationale added.
10. Discussion expanded with the chlorophyll-lifetime and fixation literature.
11. Instrument validation against a reference fluorophore (fluorescein τ recovered within 2% of nominal; four-colour test slide across all three emission channels) and segmentation validation against blind manual annotation (259 cells, three fields spanning all zones; F1 = 0.87), added as Supplementary Figures S1 and S2.
12. All figures rebuilt: unified type scale, light phasor backgrounds, panel-specific callouts, unused panels removed.
13. Conclusions rewritten as explicit hypothesis-and-test pairings.
14. Complete imaging dataset deposited (BioImage Archive S-BIAD4000, DOI 10.6019/S-BIAD4000), with measurement tables and analysis code as supplementary files.

© 2026. Published by The Company of Biologists under the terms of the Creative Commons Attribution License (https://creativecommons.org/licenses/by/4.0/).

Second decision letter

MS ID#: bio.062873R1

MS Title: Morphology and autofluorescence of the thallus of the golden-brown alga Hydrurus foetidus by optical and fluorescence-lifetime imaging

Authors: Xian Hu, Jens Ådne Rekkedal Haga, Live Lislerud Vestli, Felix Margadant and Oddmund Bakke

I have now reached a decision on the above manuscript.

The reviewer reports are shown at the bottom of this email.

As you will see, the reviewers gave very favourable reports, but Reviewer 1 has raised a few points that we would like to give you the opportunity to address prior to publication. I hope that you will be able to carry these out, because we would like to be able to accept your paper.

At this stage, we also ask you to ensure your manuscript complies with our formatting guidelines – please see our manuscript preparation guidelines for details. Provided you are able to fully address the referees' comments, we are positive about publication of your paper (we accept over 95% of revision submissions) and therefore hope you won't mind any extra work involved in reformatting your manuscript at this point.

Please upload both a 'clean' version of your Word file, along with a highlighted version clearly showing where you have made changes in the revised manuscript. Please avoid using 'Track changes' in Word files as these are lost in PDF conversion.

I should be grateful if you would also provide a point-by-point response detailing how you have dealt with the points raised by the reviewers in the 'Response to Reviewers' box. Please attend to all of the reviewers' comments. If you do not agree with any of their criticisms or suggestions please explain clearly why this is so.

Reviewer 1

Comments for the author

Thank you to the authors for the detailed responses and explanations. The manuscript has improved substantially. What concerns me is the lack of discussion connecting the results obtained with the literature context. Across the whole Results & Discussion subsection, only six manuscripts are cited, and most of these provide background specific to the alga itself. The responses letter states that relevant fixation and autofluorescence literature was cited in the Discussion in response to fixation (point 1.3), and that the chlorophyll FLIM literature was engaged with in response to point 1.8. However, the reference list provided contains no citations on fixation effects on autofluorescence, and no citations on chlorophyll photophysics or comparative FLIM studies either.

Specific comments

Introduction

"...and the species has been established in laboratory culture by others." Since only one study is cited, I would suggest replacing "by others" with the authors' names directly: "...and the species has been established in laboratory culture by Klaveness and …"

The final sentence of the Introduction ("...the statistical treatment below is structured accordingly") reads as a methodological justification. I would suggest moving this to the Methods, and closing the Introduction with the biological rationale for field sampling alone.

Results and Discussion

© 2026. Published by The Company of Biologists under the terms of the Creative Commons Attribution License (https://creativecommons.org/licenses/by/4.0/).

This section has improved and the expansion is noticeable, but it consists almost entirely of results and their interpretation. I understand that, as this is baseline research, there may be no directly comparable studies on the same organism, but there is literature available on the techniques and methodologies used. For example, in a quick search I have found FLIM-based studies in Chlamydomonas (Holub et al. 2007), Tetraselmis (Kristoffersen et al. 2016) and Chlorella (Chorvatova et al. 2020). While these algae are very different, the underlying chlorophyll a photophysics they characterise may still provide a relevant interpretive framework. It is worth noting that this was a brief search, not an exhaustive review of the literature available. The authors are better positioned to judge their relevance and to identify more appropriate citations.

Conclusions

The final paragraph repeats the construction "The proposal that... can be tested by..." three times in close succession. Varying the phrasing would improve readability without changing the content.

Reviewer 2

Comments for the author

The authors appreciated the comments of both reviewers and made significant efforts to reply.
All points are addressed correctly and sufficiently.
The manuscript now has a new, very improved face and my side finds it ready for the scientific community.
I wish authors will to continue the good work in the cold north and provide new insights after the next collection period.

Good luck with all!

Reviewer's Responses to Questions

Experimental quality
Are experiments performed using appropriate methods that will answer the question (or test the hypothesis or support the observations) posed by the authors? Is the right tool used for the job?
If 'No', please indicate reasons in Comments for Author box below.
Reviewer #1:

- Yes

Reviewer #2:

- Yes

Were the data analyzed using appropriate statistical tests?
If 'No', please indicate reasons in Comments for Author box below.
Reviewer #1:

- Yes

Reviewer #2:

- Yes

Reproducibility
Were experiments performed using adequate number of biological replicates?
If 'No', please indicate reasons in Comments for Author box below.
Reviewer #1:

- No

Reviewer #2:

- Yes

© 2026. Published by The Company of Biologists under the terms of the Creative Commons Attribution License (https://creativecommons.org/licenses/by/4.0/).

Is there sufficient raw data to assess the rigor of the analysis?
If 'No', please indicate reasons in Comments for Author box below.
Reviewer #1:
- Yes

Reviewer #2:
- Yes

Does the methods section provide sufficient detail to permit reproducibility?
If 'No', please indicate reasons in Comments for Author box below.
Reviewer #1:
- Yes

Reviewer #2:
- Yes

Completeness
Are the manuscript's conclusions supported by the data?
If 'No', please indicate reasons in Comments for Author box below.
Reviewer #1:
- Yes

Reviewer #2:
- Yes

Scholarship
Do the authors cite and discuss the merits of data that would argue for and against their conclusion?
If 'No', please indicate reasons in Comments for Author box below.
Reviewer #1:
- No

Reviewer #2:
- Yes

Does the manuscript title & abstract accurately reflect the contents of the manuscript, without hyperbole?
If 'No', please indicate reasons in Comments for Author box below.
Reviewer #1:
- Yes

Reviewer #2:
- Yes

Second revision

Author response to reviewers' comments

We thank both reviewers for their positive assessment and the editor for the opportunity to make these final changes. All changes made in this round are highlighted in yellow in the highlighted file; highlighting from the previous round has been cleared.

© 2026. Published by The Company of Biologists under the terms of the Creative Commons Attribution License (https://creativecommons.org/licenses/by/4.0/).

REVIEWER 1

Literature context. Reviewer 1 is right, and we apologise: our previous letter stated that fixation/autofluorescence and chlorophyll-FLIM literature had been added, but it had not reached the submitted manuscript. We have now added ten references on techniques, photophysics and comparative biology, each integrated into the argument rather than appended, and verified against the primary record. Specifically:

• The three suggested FLIM studies (Holub et al., 2007; Kristoffersen et al., 2016; Marcek Chorvatova et al., 2020) are now discussed where FLIM is introduced, together with why absolute lifetimes do not transfer from chlorophyll a/b chlorophytes to our chlorophyll a/c chrysophyte, and what does — the underlying chlorophyll a photophysics.

• Our two-pole interpretation is now grounded in the open/closed reaction-centre lifetime literature (Govindjee et al., 1990; Matsubara and Chow, 2004), with the caveat that our fixed, whole-cell phasor lifetimes make the comparison qualitative.

• A new paragraph addresses fixation effects on autofluorescence (Baschong et al., 2001; Joosen et al., 2014; Pegg et al., 2021), framing our lifetimes as properties of fixed material under a defined protocol and noting that the far-red channel is the least exposed to aldehyde-derived artefact.

• The mechanobiological hypotheses are placed against the macroalgal biomechanics literature (Martone et al., 2012; Starko et al., 2015), noting the difference between wave loading and steady stream flow.

In addition, our cell-size and morphology results are now compared with the earlier descriptions of wild material (Berthold, 1878; Mack, 1953; Joyon, 1963; Vesk et al., 1984) and with the cell categories of cultured material (Klaveness and Lindstrøm, 2011), whose ‘branch cells’ and ‘central axis cells’ correspond to our round and elongated cells. Results and Discussion now cites 23 works.

"...by others." Changed to "...established in laboratory culture by Klaveness and Lindstrøm (2011)".

Final sentence of the Introduction. Moved to the opening of the Statistical analysis subsection, as suggested; the Introduction now closes on the biological rationale for field sampling.

Repeated construction in the Conclusions. The three sentences now use three different constructions; content unchanged.

REVIEWER 2

We thank Reviewer 2 for the generous assessment and good wishes, and look forward to the next collection season.

ADDITIONAL CHANGES

Authorship. Kjetill Sigurd Jakobsen and Julien Alain Andre Resseguier (both Department of Biosciences, University of Oslo) have been added as co-authors: K.S.J. contributed to the conceptualization of the project and to editing the manuscript, and J.A.A.R. to the optimization of the initial fixation protocol and to editing the manuscript. All authors have seen and approved the revised manuscript and the change in authorship.

Figure 1. Panel B now shows an overview of the collection site (photographed on an earlier visit, as stated in the legend), with the collection-day sampling photograph as an inset; text previously overlaid on panels B and C has been moved to the legend.

Terminology and presentation. Cell-type terms are used consistently throughout (‘stipe’ and ‘medullary’ replaced by ‘central axis’), and scientific names are italicised. The genome-resources sentence in the Introduction now cites the two recent long-read assemblies (Francomme, 2025; GenBank acc. no. GCA_978795795.1), and the Data and Resource Availability statement cites the BioStudies database (Sarkans et al., 2017).

© 2026. Published by The Company of Biologists under the terms of the Creative Commons Attribution License (https://creativecommons.org/licenses/by/4.0/).

Collection date. The collection date has been corrected from 17 to 16 February 2026 (Materials and Methods, Statistical analysis and Fig. 1), confirmed against the time-stamped sampling photograph.

Figure 2 and supplementary figures. Figure 2 labels now use the 'round' and 'elongated' terms of the text. The P value shown in Fig. 2D has been corrected to the two-sided value (6.9×10^{-5}) already given in the text and legend; the panel previously showed the one-sided value, and the data are unchanged. The legends of Figs S1 and S2 have been rewritten for clarity, with no change to the data.

FORMATTING

Word count 5,006 (Introduction and Results and Discussion 4,035 + figure legends 971; limit 8000; title page, abstract, Materials and Methods and references excluded as specified). Four display items. Clean and highlighted Word files supplied with identical content; no Track Changes used. Supplementary material is a single collated PDF (4 pages, 5.3 MB) containing Figs S1–S2 and Table S1, with no text sections. Submission checklist completed.

Third decision letter

MS ID#: bio.062873R2

MS Title: Morphology and autofluorescence of the thallus of the golden-brown alga Hydrurus foetidus by optical and fluorescence-lifetime imaging

Authors: Xian Hu, Jens Ådne Rekkedal Haga, Live Lislerud Vestli, Kjetill Sigurd Jakobsen, Julien Alain Andre Resseguier, Felix Margadant and Oddmund Bakke

I am happy to tell you that your manuscript has been accepted for publication in Biology Open, pending our standard publication integrity checks. It was accepted on 30th September 2026.

© 2026. Published by The Company of Biologists under the terms of the Creative Commons Attribution License (https://creativecommons.org/licenses/by/4.0/).
